# Dual targeting macrophages and microglia is a therapeutic vulnerability in models of *PTEN*-deficient glioblastoma

**DOI:** 10.1172/JCI178628

**Published:** 2024-10-01

**Authors:** Yang Liu, Junyan Wu, Hinda Najem, Yiyun Lin, Lizhi Pang, Fatima Khan, Fei Zhou, Heba Ali, Amy B. Heimberger, Peiwen Chen

**Affiliations:** 1 Department of Neurological Surgery, Feinberg School of Medicine, Northwestern University, Chicago, Illinois, USA.; 2 Department of Cancer Biology, Lerner Research Institute, Cleveland Clinic, Cleveland, Ohio, USA.; 3 Department of Genetics and; 4 UTHealth Graduate School of Biomedical Sciences, The University of Texas MD Anderson Cancer Center, Houston, Texas, USA.; 5 Case Comprehensive Cancer Center, Cleveland, Ohio, USA.

**Keywords:** Oncology, Brain cancer, Cancer immunotherapy, Macrophages

## Abstract

Tumor-associated macrophages and microglia (TAMs) are critical for tumor progression and therapy resistance in glioblastoma (GBM), a type of incurable brain cancer. We previously identified lysyl oxidase (LOX) and olfactomedin like-3 (OLFML3) as essential macrophage and microglia chemokines, respectively, in GBM. Here, single-cell transcriptomics and multiplex sequential immunofluorescence followed by functional studies demonstrate that macrophages negatively correlate with microglia in the GBM tumor microenvironment. LOX inhibition in *PTEN*-deficient GBM cells upregulates OLFML3 expression via the NF-κB-PATZ1 signaling pathway, inducing a compensatory increase of microglia infiltration. Dual targeting macrophages and microglia via inhibition of LOX and the CLOCK-OLFML3 axis generates potent antitumor effects and offers a complete tumor regression in more than 60% of animals when combined with anti-PD1 therapy in *PTEN*-deficient GBM mouse models. Thus, our findings provide a translational triple therapeutic strategy for this lethal disease.

## Abstract 2

<strong>Dual targeting macrophages and microglia is a therapeutic vulnerability in models of *PTEN*-deficient glioblastoma</strong>

## Graphical abstract

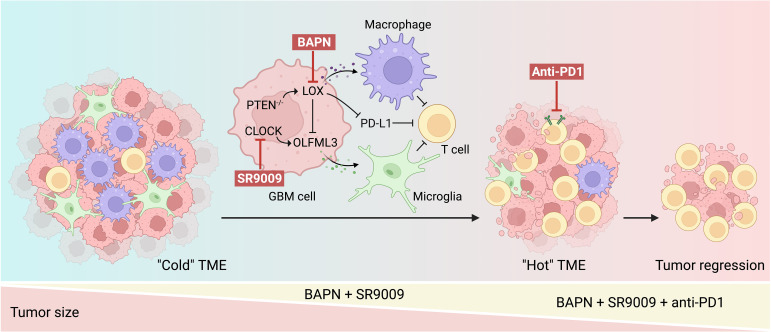

## Introduction

Glioblastoma (GBM) is inevitably fatal and the most aggressive type of brain tumor in adults, with a 5-year survival rate of approximately 10% (1, 2). Although marked progress has been achieved in understanding GBM pathogenesis, the prognosis of patients with GBM remains dismal and the median overall survival is still only 15–20 months after initial diagnosis (3–6). The current standard of care for GBM includes maximal safe surgical resection followed by radiation with concurrent temozolomide and adjuvant temozolomide with the unfortunate development of treatment resistance (7–9). In-depth studies of GBM genomics have yielded detailed atlases of oncogene and tumor suppressor gene alterations (7, 10–12). *PTEN* loss occurs in about 30%–40% of patients with GBM and 80%–90% of patients with GBM harbor alterations of the receptor tyrosine kinase (RTK)/PI3K/PTEN pathway (10). Our recent studies have also identified *CLOCK* as a potential oncogene that is amplified in about 5% of GBM cases (13). Despite the substantial contribution of known genetic drivers in promoting GBM development, targeted therapies such as those against RTK signaling have failed in the clinic due to the intratumoral heterogeneity, which ensures the survival of subpopulations of GBM cells in treated tumors (7, 14).

Increasing evidence shows that aberrant cancer-associated molecular activities that result from gene alterations are not limited to cancer cells, but also extend to stromal cells in the tumor microenvironment (TME) (15–17). Tumor-associated macrophages and microglia (TAMs) are the most prominent immune cell populations in the TME, which account for up to 50% of total cells in the GBM tumor mass (18, 19). We have shown that *PTEN* inactivation and CLOCK overexpression in GBM cells upregulate lysyl oxidase (LOX) and olfactomedin like 3 (OLFML3), which trigger the infiltration of macrophages and microglia, respectively, into the GBM TME (13, 20). Inhibition of LOX and CLOCK-OLFML3 axis markedly inhibits tumor growth and suppresses macrophage and microglia infiltration in GBM mouse models (13, 20), indicating that targeting LOX and CLOCK-OLFML3 axis are promising therapeutic strategies for reducing the infiltration of immunosuppressive and tumor potentiating macrophages and microglia into the GBM TME. However, the understanding of the functional relationship between macrophages and microglia in GBM is limited.

Immunotherapies, including immune checkpoint inhibitor (ICI) therapies, have been shown to improve patient outcomes in multiple cancer types (21, 22). However, emerging evidence demonstrates that such ICI therapies only produce modest clinical benefit in patients with GBM due to the presence of highly immunosuppressive cells (e.g., TAMs) in the TME (23–27). Genomic and transcriptomic analyses in tumors of patients with GBM have shown that the presence of *PTEN* mutations and higher macrophage abundance are associated with the lack of response to anti-PD1 therapy (28), suggesting that TAMs might contribute to the development of anti-PD1 therapy resistance in *PTEN*-deficient GBM. On the other hand, TAMs have been shown to negatively impact the antitumor response of conventional therapies, such as radiotherapy (29, 30). These findings support the importance of TAMs in affecting therapy resistance; however, there is no effective therapeutic approach to target them in the GBM TME.

In this study, we reveal that macrophages are negatively correlated with microglia in the GBM TME. Specifically, suppressing macrophage infiltration in *PTEN*-deficient GBM via LOX inhibition upregulates the expression of OLFML3 in GBM cells, which induces a compensatory increase of microglia infiltration into the TME. We hypothesized that blockade of macrophage infiltration and its compensatory effect on microglia may result in a robust antitumor effect, which might be augmented when combined with anti-PD1 therapy in *PTEN*-deficient GBM mouse models. Our preclinical trials confirm that the triple therapy (LOX inhibition + CLOCK-OLFML3 axis blockade + anti-PD1 therapy) leads to disease eradication in more than 60% of GBM-bearing mice.

## Results

### Targeting LOX improves the efficacy of anti-PD1 therapy in PTEN-deficient GBM.

**Figure 1:**
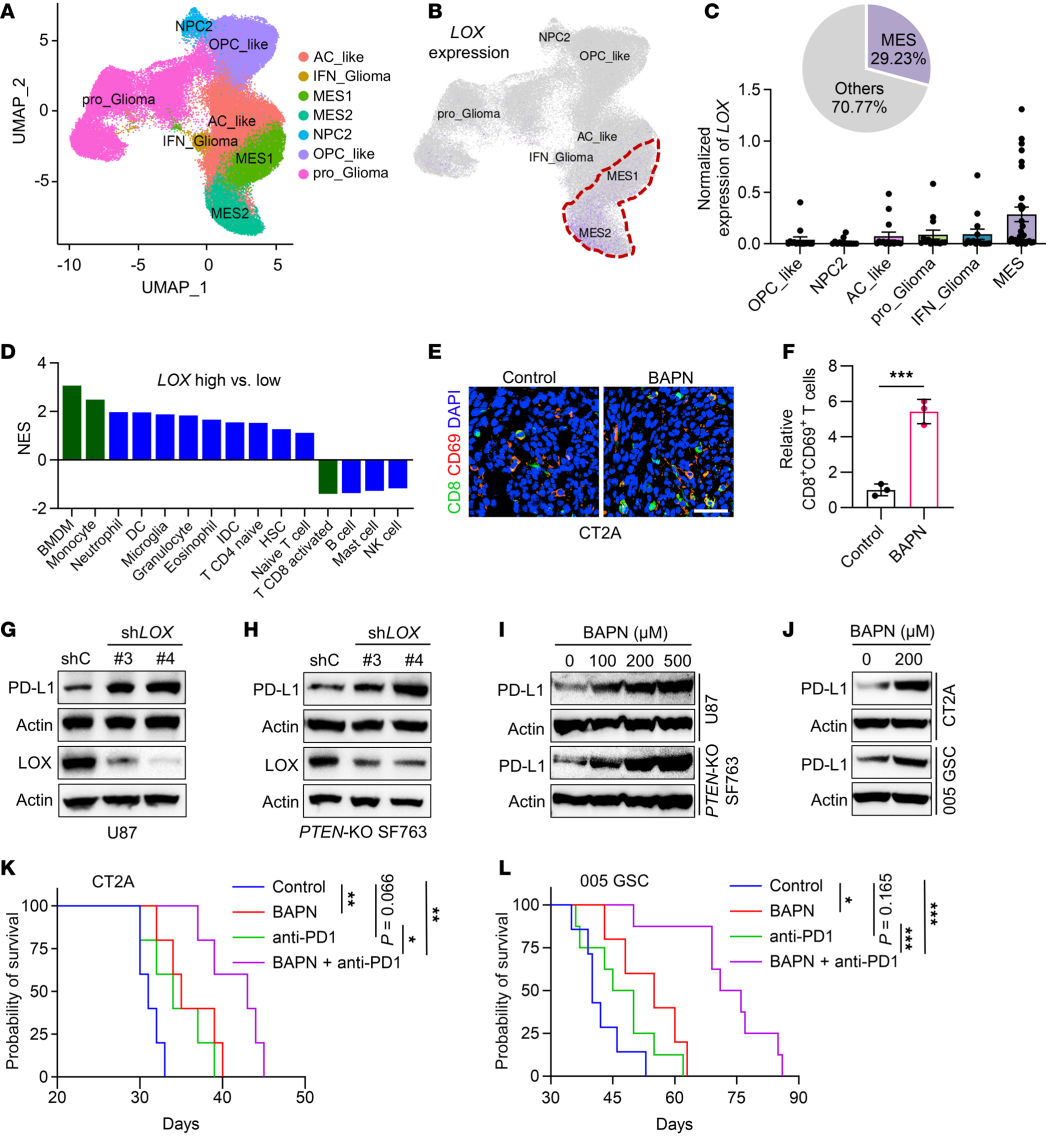
LOX inhibition improves the efficacy of anti-PD1 therapy. (**A**) High-resolution uniform manifold approximation and projection (UMAP) dimensional reduction of different subtypes, including mesenchymal-like (MES-like), neural-progenitor-like (NPC-like), astrocyte-like (AC-like) and oligodendrocyte-progenitor-like (OPC-like), of tumor cells from tumors from patients with GBM based on the scRNA-seq dataset (GSE182109). (**B**) Pattern representing single-cell gene expression of *LOX* in distinct subtypes of tumor cells based on above scRNA-seq dataset. (**C**) Percentage MES-like GBM cells out of total GBM cells, and normalized *LOX* gene expression in different subtypes of malignant cells in tumors from patients with GBM based on above scRNA-seq dataset. (**D**) GSEA analysis for various types of immune cells in *LOX*-high (*n* = 123) and *LOX*-low (*n* = 122) patient tumors from the TCGA GBM database. (**E** and **F**) IF (**E**) and quantification (**F**) of relative CD8^+^CD69^+^ T cells in tumors from CT2A tumor-bearing mice treated with or without LOX inhibitor BAPN (2 g/L in drinking water) on day 4. Scale bar: 50 μm. *n* = 3 independent samples. Student’s *t* test. (**G** and **H**) Immunoblots for PD-L1 and LOX in lysates of U87 (**G**) and *PTEN*-KO SF763 (**H**) cells expressing shRNA control (shC) and *LOX* shRNAs (sh*LOX*). (**I**) Immunoblots for PD-L1 in lysates of U87 and *PTEN*-KO SF763 cells treated with BAPN at indicated concentrations. (**J**) Immunoblots for PD-L1 in lysates of CT2A cells and 005 GSCs treated with BAPN at indicated concentration. (**K** and **L**) Survival curves of C57BL/6J mice implanted with CT2A cells (2 × 10^4^ cells/mouse, **K**) or 005 GSCs (2 × 10^5^ cells/mouse, **L**). Mice were treated with BAPN (2 g/L in drinking water) on day 4, and then received the treatment with IgG or anti-PD1 (10 mg/kg body weight, i.p.) on days 11, 14, and 17. *n* = 5–7 mice per group. Log-rank test. *P < 0.05; **P < 0.01; ***P < 0.001.

Our previous studies revealed that macrophage chemokine LOX is upregulated in *PTEN*-deficient GBM cells (20). To confirm the expression pattern of LOX, we analyzed the single-cell RNA sequencing (scRNA-seq) data from tumors of patients with GBM (31) with results showing that *LOX* was highly expressed in mesenchymal GBM cells, which account for 29.23% of total malignant cells (Figure 1, A–C). *PTEN* deficiency is common in mesenchymal GBM subtype, which harbors higher immunosuppressive macrophages relative to classical and proneural GBMs (20, 32). To identify specific immune cells linked to LOX expression in GBM, we audited the TCGA GBM tumors for 15 types of immune cells with validated gene set signatures (13, 20). Bone marrow–derived macrophages (BMDMs) and monocytes were identified as the top immune cell types enriched in *LOX*-high tumors compared to *LOX*-low tumors. Conversely, an activated CD8^+^ T cell signature was reduced in *LOX*-high tumors (Figure 1D). These findings suggest a potential connection between LOX-regulated macrophages and CD8^+^ T cells in GBM, which promoted us to explore the role of LOX inhibition in regulating antitumor immune responses in *PTEN*-deficient GBM mouse models.

To confirm its role in regulating immune response in vivo, we developed GBM mouse models by intracranial injection of CT2A (*PTEN*-deficient) or 005 GSC, a GSC line harboring activated AKT (33, 34), and treated them with LOX neutralizing antibodies or LOX inhibitor β-aminopropionitrile (BAPN), which showed an ability to cross the blood-brain barrier (BBB) (Supplemental Figure 1, A–C; supplemental material available online with this article; https://doi.org/10.1172/JCI178628DS1). Immunofluorescence (IF) staining demonstrated that treatment with BAPN or LOX-neutralizing antibodies in tumor-bearing C57BL/6J mice increased intratumoral CD8^+^ T cells (Supplemental Figure 1, D–I) and activated CD8^+^ (CD8^+^CD69^+^) T cells (Figure 1, E and F). Given the role of PD-L1 in regulating immunosuppression in GBM, we investigated whether LOX affects the expression of PD-L1 in *PTEN*-deficient GBM cells (e.g., U87, CT2A, and *PTEN* CRISPR-KO SF763 cells) and GSCs (e.g., 005 GSC, GSC23, and GSC7-10) (20). The results showed that LOX inhibition genetically (e.g., shRNA-mediated LOX depletion) and pharmacologically (treatment with LOX inhibitor BAPN) upregulated the expression of PD-L1 in *PTEN*-deficient GBM cells (Figure 1, G–J and Supplemental Figure 1, J–L). Together, these findings led us to hypothesize that LOX inhibition could improve the efficacy of anti-PD1 therapy in *PTEN*-deficient GBM mouse models. Indeed, our results showed that BAPN treatment extended survival of mice bearing CT2A and 005 GSC tumors, and the antitumor effect was further augmented when BAPN was combined with anti-PD1 therapy (Figure 1, K and L).

### The negative association between macrophages and microglia in the GBM TME.

**Figure 2:**
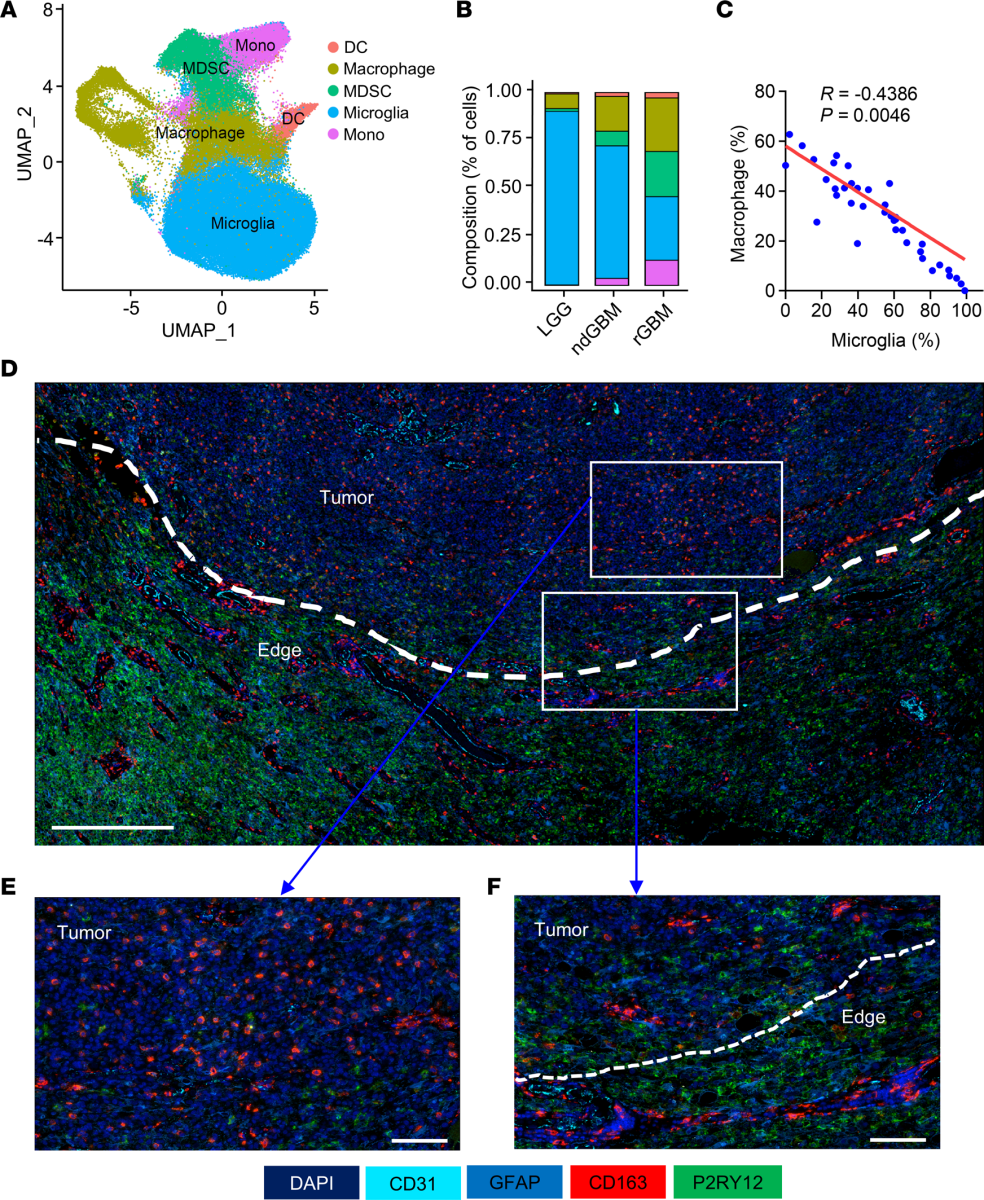
Macrophages negatively related to microglia in GBM tumors. (**A**) High-resolution UMAP dimensional reduction of myeloid cells, including macrophages, microglia, monocytes (Mono), dendritic cells (DCs), and myeloid-derived suppressor cells (MDSCs), from tumors from patients with GBM based on the scRNA-seq dataset (GSE182109). (**B**) Percentage of different types of myeloid cells in tumors of low-grade gliomas (LGG), newly diagnosed GBM (ndGBM) and recurrent GBM (rGBM) based on above scRNA-seq data. (**C**) Correlation between macrophages and microglia in the GBM TME based on the above scRNA-seq data. Pearson test. (**D**) Representative image of multiplex sequential immunofluorescence showing the distribution of P2RY12^+^ microglia, CD163^+^ macrophages, GFAP^+^ tumor cells, and CD31^+^ blood vessels in the tumor edges and tumors from IDH1-WT GBM patients. Scale bar: 500 μm. (**E** and **F**) Higher magnified view of CD163^+^ macrophages and P2RY12^+^ microglia in the tumor edges and tumors from IDH1-WT GBM patients. Scale bar: 100 μm.

Although our studies demonstrated that LOX inhibition alone and in combination with anti-PD1 therapy can inhibit GBM progression, no mice cleared their tumors after the treatment (Figure 1, K and L). We hypothesized that LOX inhibition–induced impairment of macrophage infiltration might induce a compensatory change of other immune cells in the GBM TME. To test this, we analyzed the scRNA-seq data (31) from glioma patient tumors with a focus on myeloid cells, which include macrophages, microglia, monocytes, dendritic cells (DCs) and myeloid-derived suppressor cells (MDSCs). Among them, macrophages and microglia are the dominant cell populations (Figure 2A). By analyzing these myeloid cells in low-grade gliomas (LGG), newly diagnosed GBM (ndGBM), and recurrent GBM (rGBM), we found that macrophage/monocyte density was very low in LGG, increased in ndGBM, and was highly enriched in rGBM, whereas microglia showed the opposite expression pattern (Figure 2B), suggesting a negative correlation between them in tumors of patients with glioma. Further analysis in tumor samples of patients with GBM revealed that the macrophage abundance was negatively correlated with microglia in the TME (Figure 2C and Supplemental Figure 2, A and B). Next, we performed multiplex sequential immunofluorescence (SeqIF) to stain and image whole mount sections of tumors from patients with IDH1-WT GBM in continuity with the adjacent brain parenchyma (*n* = 3). The results showed that P2RY12^+^ microglia were mostly distributed in the parenchyma and GBM margin, whereas CD163^+^ macrophages were highly enriched in the tumors (Figure 2D and Supplemental Figure 2C). Higher magnified view of tumor sections demonstrated that CD163^+^ macrophages were distributed in the perivascular niches in the tumor and at the brain interface (Supplemental Figure 2, D and E). More specifically, in densely cellular tumor regions, P2RY12^+^ microglia were absent when tumors harbor high abundance of CD163^+^ macrophages (Figure 2E). Conversely, CD163^+^ macrophages were relatively low when tumors have high infiltration of P2RY12^+^ microglia (Figure 2F).

### LOX inhibition reduces macrophage infiltration but upregulates OLFML3 expression and microglia infiltration in GBM.

**Figure 3:**
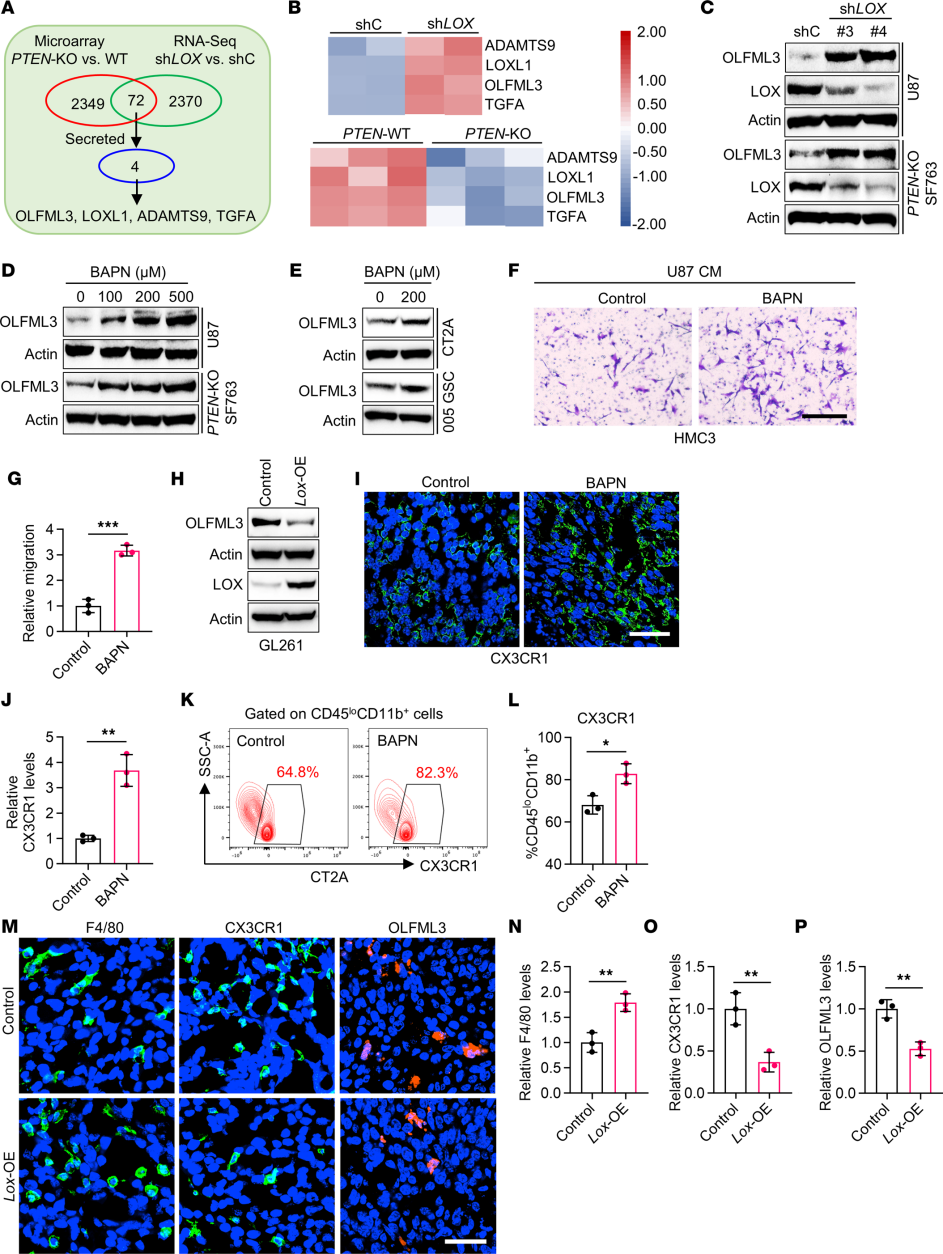
LOX negatively regulates OLFML3 expression and microglia infiltration in GBM. (**A** and **B**) Identification (**A**) and expression heatmap (**B**) of 4 overlapping PTEN-LOX axis-regulated genes encoding secreted factors in *PTEN*-KO versus WT SF763 cells and in *LOX* shRNA (sh*LOX*) versus shRNA control (shC) U87 cells. Red signal indicates higher expression and blue signal denotes lower expression. (**C**) Immunoblots for OLFML3 and LOX in lysates of U87 and *PTEN*-KO SF763 cells expressing shC and sh*LOX*. (**D** and **E**) Immunoblots for OLFML3 in lysates of U87 and *PTEN*-KO SF763 cells (**D**) and CT2A cells and 005 GSCs (**E**) treated with BAPN at indicated concentrations. (**F** and **G**) Representative images (**F**) and quantification (**G**) of relative migration of HMC3 microglia following stimulation with the conditioned media (CM) from U87 cells pretreated with or without BAPN (200 μM). Scale bar: 400 μm. *n* = 3 independent samples. Student’s *t* test. (**H**) Immunoblots for OLFML3 and LOX in lysates of GL261 cells in the presence or absence of LOX overexpression (OE). (**I** and **J**) IF (**I**) and quantification (**J**) of relative CX3CR1^+^ microglia (green) in tumors from CT2A-bearing mice treated with or without BAPN (2 g/L in drinking water) on day 4. DAPI (blue). Scale bar: 50 μm. *n* = 3 independent samples. Student’s *t* test. (**K** and **L**) Representative images (**K**) and quantification (**L**) of flow cytometry for the percentage of intratumoral CD45^lo^CD11b^+^CX3CR1^+^ microglia in size-matched tumors from CT2A tumor-bearing mice treated with or without BAPN. *n* = 3 independent samples. Student’s *t* test. (**M**–**P**) IF (**M**) and quantification of relative F4/80^+^ macrophages (**N**, green), CX3CR1^+^ microglia (**O**, green), and OLFML3^+^ cells (**P**, red) in tumors from mice implanted with control and LOX-overexpressed GL261 cells. DAPI (blue). Scale bar: 50 μm. *n* = 3 independent samples. Student’s t test. *P < 0.05; **P < 0.01; and ***P < 0.001.

Given the critical role of the PTEN-LOX signaling axis in triggering macrophage infiltration (20), we investigated whether LOX inhibition can induce compensatory changes of chemokines that might affect microglia infiltration. To this end, we performed RNA-seq profiling in U87 cells with *LOX* shRNA (sh*LOX*) versus shRNA control (shC). By analyzing the RNA-seq data as well as microarray data of SF763 cells with *PTEN*-KO versus WT (20), we identified 4 genes (*OLFML3*, *LOXL1*, *ADAMTS9*, and *TGFA*) that were upregulated by LOX knockdown and downregulated by *PTEN* KO in GBM cells (Figure 3, A and B). Among them, OLFML3 attracted our attention since our previous studies showed that OLFML3 is a microglia chemokine in GBM (13, 35). Immunoblotting results confirmed that shRNA-mediated LOX knockdown in *PTEN*-deficient GBM cells (e.g., U87 and *PTEN*-KO SF763 cells) and GSCs (e.g., GSC23 and GSC7-10) upregulated OLFML3 expression (Figure 3C and Supplemental Figure 3, A and B). Similarly, pharmacologic inhibition of LOX using the inhibitor BAPN increased the expression of OLFML3 in both human (e.g., U87, *PTEN*-KO SF763, GSC23, and GSC7-10) and mouse (e.g., CT2A and 005 GSCs) GBM cells and GSCs (Figure 3, D and E and Supplemental Figure 3C).

To confirm whether LOX inhibition–induced upregulation of OLFML3 could affect microglia infiltration in the GBM TME, we first performed transwell migration assays with results showing that the conditioned media (CM) from LOX-depleted or inhibited U87 cells increased the migration ability of HMC3 microglia (Figure 3, F and G and Supplemental Figure 3, D and E). Next, we overexpressed LOX in *PTEN*-WT GL261 cells (Figure 3H) and then checked OLFML3 expression in control and LOX-overexpressed (*Lox*-OE) cells and used CM from them to perform transwell migration assay. The results showed that LOX overexpression downregulated OLFML3 expression in GBM cells (Figure 3H) and reduced the migration of SIM-A9 microglia (Supplemental Figure 3, F and G). In addition to these in vitro studies, we analyzed microglia and macrophage populations in control, LOX-inhibited, and LOX-overexpressed tumors. The results from IF staining and flow cytometry showed that BAPN-treated CT2A tumors had higher CX3CR1^+^ (IF) and CD45^lo^CD11b^+^CX3CR1^+^ (flow cytometry) microglia (Figure 3, I–L) and lower F4/80^+^ (IF) and CD45^hi^CD11b^+^CD68^+^ (flow cytometry) macrophages (Supplemental Figure 3, H–K) compared with control tumors. In contrast, LOX overexpression induced higher infiltration of macrophages and lower OLFML3 expression and microglia infiltration in the GBM TME (Figure 3, M–P). To further confirm whether this effect is dependent on GBM cells or direct macrophage-microglia interaction, we used clodronate liposomes to deplete macrophages in tumor-bearing mice. The results showed that LOX overexpression in GL261 cells still downregulated OLFML3 expression and microglia infiltration in macrophage-depleted tumors (Supplemental Figure 3, L–O).

**Figure 4:**
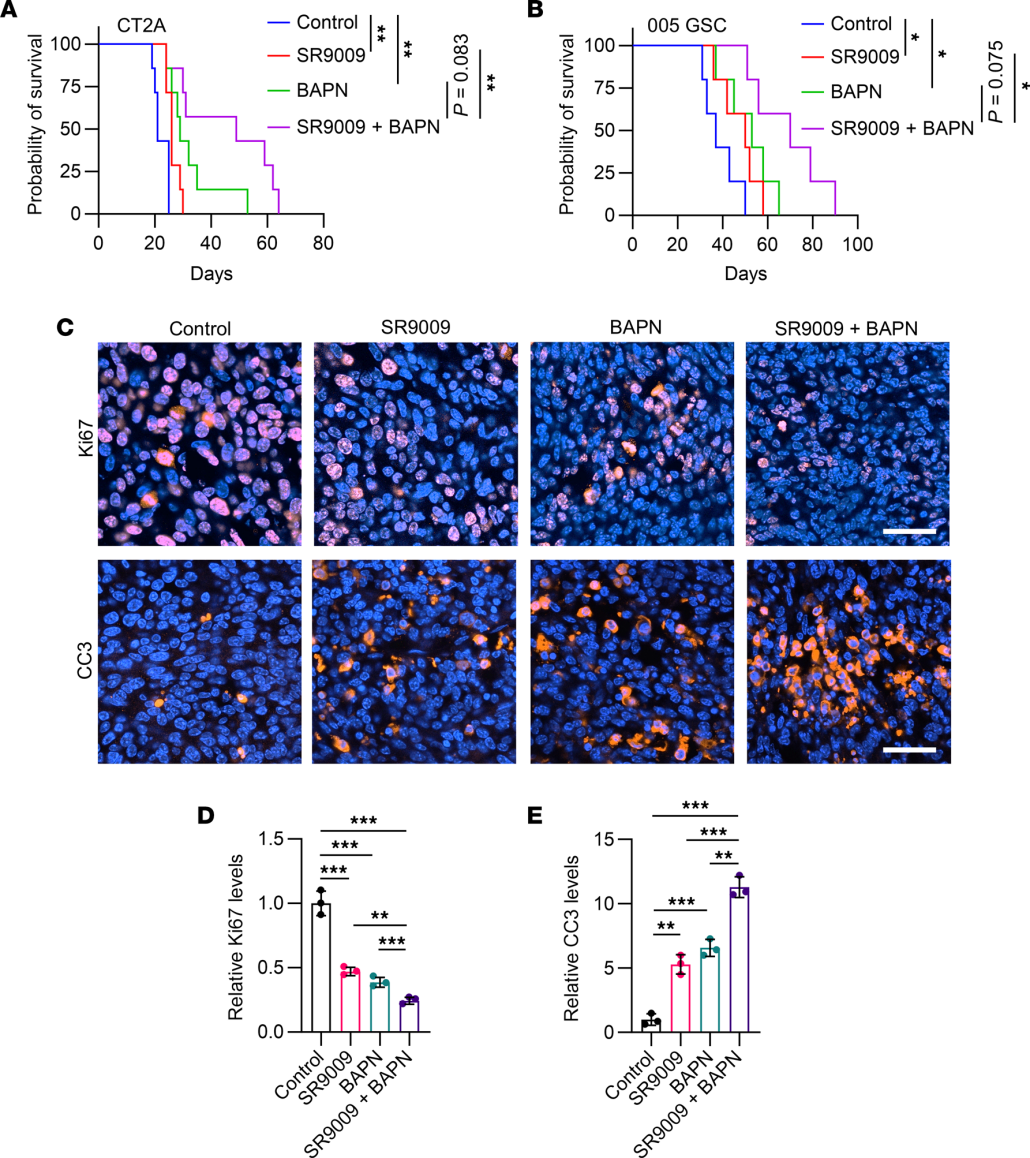
Dual Inhibition of LOX and CLOCK-OLFML3 axis exhibits a potent antitumor effect in GBM mouse models. (**A** and **B**) Survival curves of C57BL/6J mice implanted with CT2A cells (2 × 10^4^ cells/mouse, **A**) or 005 GSCs (2 × 10^5^ cells/mouse, **B**). Mice were treated with BAPN on day 4, and/or SR9009 (100 mg/kg/day, i.p) for 10 days beginning at day 7. *n* = 5–7 mice per group. Log-rank test. (**C**–**E**) Representative (**C**) and quantification (**D** and **E**) of immunofluorescence staining of Ki67 and cleaved caspase 3 (CC3) in tumors from CT2A-bearing mice treated with or without BAPN and SR9009. Scale bars: 50 μm. *n* = 3 independent samples. 1-way ANOVA test. *P < 0.05; **P < 0.01; and ***P < 0.001.

Next, we aimed to examine whether inhibition of CLOCK-mediated OLFML3 expression and microglia infiltration will affect LOX expression and macrophage biology. Immunoblotting results showed that shRNA-mediated depletion of CLOCK and pharmacologic inhibition of CLOCK using the Rev-ErbA agonist SR9009 did not affect the expression of LOX in QPP7 GSCs (*PTEN*-deficient), CT2A cells, and 005 GSCs (Supplemental Figure 4, A–C). Accordingly, the CM from SR9009-treated U87 cells did not change the migration ability of THP-1 macrophages (Supplemental Figure 4, D and E). To confirm it in vivo, we developed GBM mouse model and confirmed that SR9009 can cross the BBB (Supplemental Figure 4, F–H). Consistent with our previous studies (13, 35), we found that inhibition of CLOCK using SR9009 reduced CX3CR1^+^ microglia (Supplemental Figure 4, I and J). However, SR9009 treatment did not affect F4/80^+^ macrophages (Supplemental Figure 4, K and L). As evidenced by our recent publications (13, 20), here, we further confirmed that SR9009 treatment impaired GBM cell proliferation, but LOX inhibition using BAPN had no such effect (Supplemental Figure 4, M–S). Together, these findings encouraged us to develop an effective therapeutic strategy by targeting the compensatory mechanism between macrophages and microglia via simultaneously inhibiting LOX and the CLOCK-OLFML3 axis. When we conducted the proof-of-principal combination of BAPN and SR9009 in GBM-bearing mice, we observed a significant survival extension relative to monotherapy in both CT2A and 005 GSC models (Figure 4, A and B). On the histological level, proliferation marker Ki67 was dramatically decreased, whereas apoptosis marker cleaved caspase 3 (CC3) was significantly increased in BAPN and SR9009 combination treatment group compared with single treatment and control groups (Figure 4, C–E).

### LOX affects OLFML3 expression via regulating the NF-κB-PATZ1 signaling axis.

**Figure 5:**
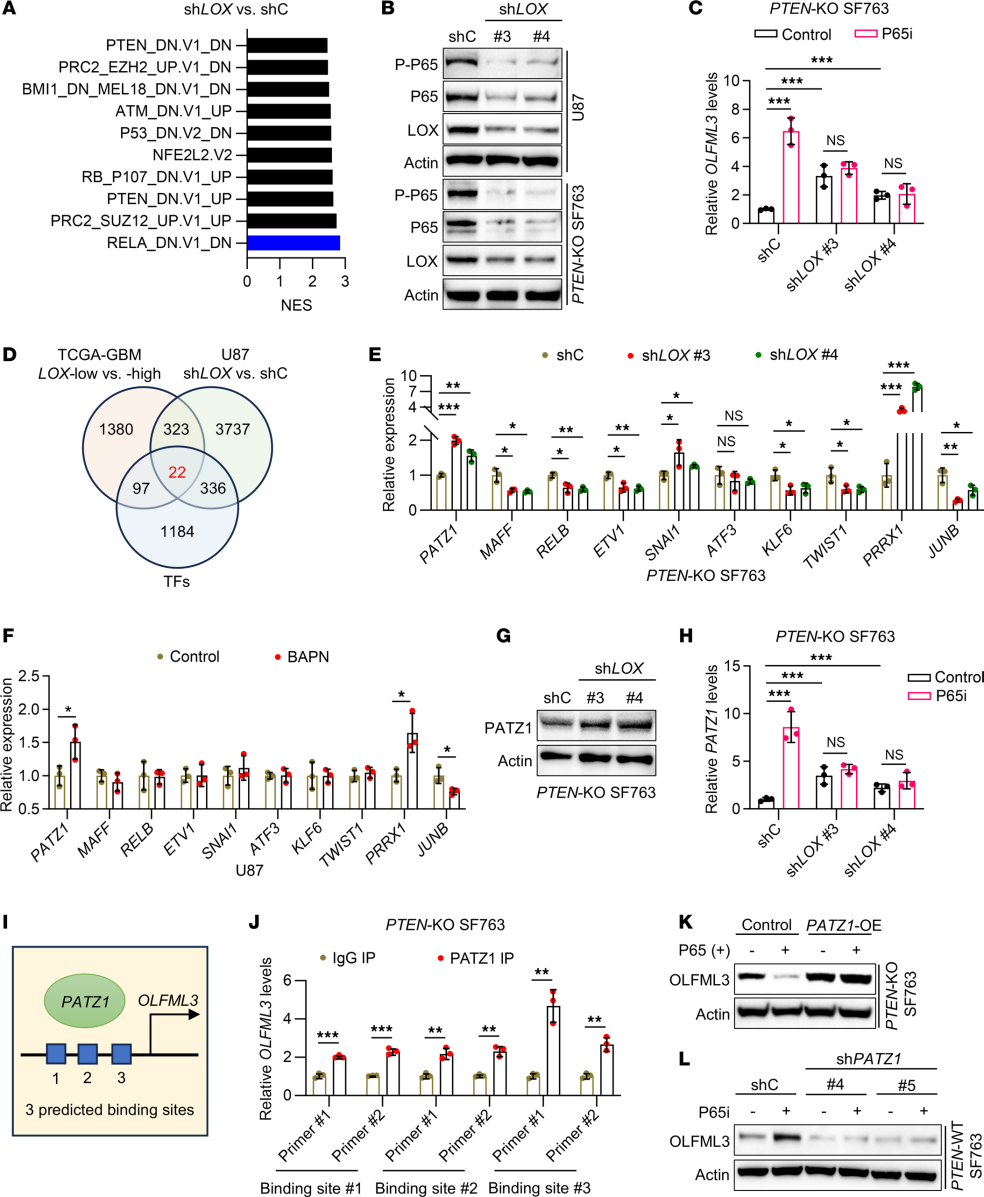
LOX regulates OLFML3 expression through regulating the NF-κB-PATZ1 signaling axis. (**A**) GSEA analysis on RNA-seq data of U87 cells with *LOX* shRNA knockdown (sh*LOX*) versus shRNA control (shC) shows top 10 enriched oncogenic signaling pathways. (**B**) Immunoblots for P-P65, P65, and LOX in lysates of U87 and *PTEN*-KO SF763 cells expressing shC and sh*LOX*. (**C**) Relative mRNA expression of *OLFML3* in *PTEN*-KO SF763 cells expressing shC and sh*LOX* treated with or without P65 inhibitor (P65i) SC75741 (5 μM). *n* = 3 independent samples. Student’s *t* test. (**D**) Identification of 22 overlapping transcription factors (TFs) in TCGA GBM tumors (*LOX-*low versus -high) and U87 cells (sh*LOX* versus shC). (**E**) Relative mRNA expression of 10 TFs in *PTEN*-KO SF763 cells expressing shC and sh*LOX*. *n* = 3 independent samples. 1-way ANOVA test. (**F**) Relative mRNA expression of the 10 TFs in U87 cells treated with or without LOX inhibitor BAPN (200 μM). *n* = 3 independent samples. Student’s *t* test. (**G**) Immunoblots for PATZ1 in lysates of *PTEN*-KO SF763 cells expressing shC and sh*LOX*. (**H**) Relative mRNA expression of *PATZ1* in *PTEN*-KO SF763 cells expressing shC and sh*LOX* and treated with or without P65i SC75741 (5 μM). *n* = 3 independent samples. Student’s *t* test. (**I**) Schematic of designing ChIP-qPCR primers based on 3 potential binding sites. (**J**) Quantification of PATZ1 ChIP-qPCR in the *OLFML3* promoter of *PTEN*-KO SF763 cells. IgG was used as the control. *n* = 3 independent samples. Student’s *t* test. (**K**) Immunoblots for OLFML3 in lysates of *PTEN*-KO SF763 cells with or without PATZ1 overexpression (OE) and treated with or without P65 activator (+). (**L**) Immunoblots for OLFML3 in lysates of *PTEN*-WT SF763 cells expressing shC and sh*PATZ1* treated with or without P65i SC75741 (5 μM). *P < 0.05; **P < 0.01; ***P < 0.001; n.s., not significant (*P* > 0.05).

To explore the potential mechanism for how LOX regulates OLFML3, we used GSEA to catalog oncogenic signaling pathways modulated by LOX in U87 cells (sh*LOX* versus shC). The RELA_DN.v1_DN was identified as the top signature affected by LOX (Figure 5A and Supplemental Figure 5A), suggesting the importance of LOX in regulating NF-κB pathway. The results from immunoblotting demonstrated that shRNA-mediated depletion of LOX in U87 cells, *PTEN*-KO SF763 cells, GSC23, and GSC7-10 significantly inhibited the NF-κB subunit P65 and Phospho-P65 (Figure 5B and Supplemental Figure 5B). To investigate the potential functional relevance of P65 in regulating OLFML3 expression in GBM cells, we treated shC and sh*LOX* *PTEN*-KO SF763 cells with the P65 inhibitor SC75741. The results showed that inhibition of P65 upregulated the expression of *OLFML3* in shC cells, but not in sh*LOX* cells (Figure 5C).

To further identify LOX-regulated factors that can transcriptionally regulate OLFML3 in *PTEN*-null GBM cells, we overlapped the differential expressed genes encoding human transcriptional factors (TFs) in U87 cells with sh*LOX* versus shC and in TCGA GBM tumors with *LOX-*low versus *LOX*-high expression. As a result, 22 potential TFs were identified (Figure 5D), which were inserted into the JASPAR database (36) with results showing that 10 of them can potentially bind to the *OLFML3* promoter. The results from RT-qPCR assays in *PTEN*-null GBM cells, such as U87, *PTEN*-KO SF763, and U251 cells, revealed that *PATZ1* and *PRRX1* were upregulated upon shRNA-mediated LOX depletion and the treatment with LOX inhibitor BAPN (Figure 5, E and F and Supplemental Figure 5, C and D). Bioinformatics analyses in TCGA of tumors of patients with GBM demonstrated that *PATZ1* correlated negatively with *LOX*, whereas *PRRX1* showed a positive correlation with *LOX* (Supplemental Figure 5, E and F). The results from immunoblotting confirmed that depletion of LOX upregulated PATZ1 protein level in *PTEN*-KO SF763 cells and *PTEN*-deficient GSC23 and GSC7-10 cells (Figure 5G and Supplemental Figure 5G). Next, we aimed to confirm whether *PATZ1* is regulated by P65 and whether PATZ1 can bind to the promotor of *OLFML3* in *PTEN*-null GBM cells. RT-qPCR demonstrated that P65 inhibition upregulated the expression of *PATZ1* in shC, but not in sh*LOX PTEN*-KO SF763 cells (Figure 5H), suggesting that PATZ1 is a downstream TF of the NF-κB pathway. Based on the predicted binding sites (Figure 5I), we designed 6 pairs of primers and performed ChIP-PCR assays with results showing that PATZ1 bound to the *OLFML3* promoter in *PTEN*-KO SF763 cells (Figure 5J). To further validate the function of NF-κB-PATZ1 signaling axis in regulating OLFML3 in GBM cells, we overexpressed PATZ1 in *PTEN*-KO SF763 cells (Supplemental Figure 5H) and found that PATZ1 overexpression enhanced OLFML3 expression and abolished P65 activation–induced downregulation of OLFML3 (Figure 5K). Conversely, shRNA-mediated PATZ1 depletion in *PTEN*-WT SF763 cells negated P65 inhibition-induced upregulation of OLFML3 (Figure 5L and Supplemental Figure 5I). Given that our previous studies have shown that OLFML3 can be transcriptionally regulated by CLOCK in GBM, we investigated whether the regulatory effect of LOX-NF-κB-PATZ1 signaling axis on OLFML3 transcription was independent of CLOCK. Immunoblotting results showed that LOX or P65 inhibition–induced OLFML3 upregulation was rescued by the treatment with SR9009 (Supplemental Figure 5, J and K). Together, these findings suggest that inhibition of LOX upregulates OLFML3 via regulating the NF-κB-PATZ1 signaling axis in *PTEN*-null GBM cells.

### Dual inhibition of LOX and CLOCK-OLFML3 axis activates antitumor immune response and synergizes with anti–PD1 therapy.

**Figure 6:**
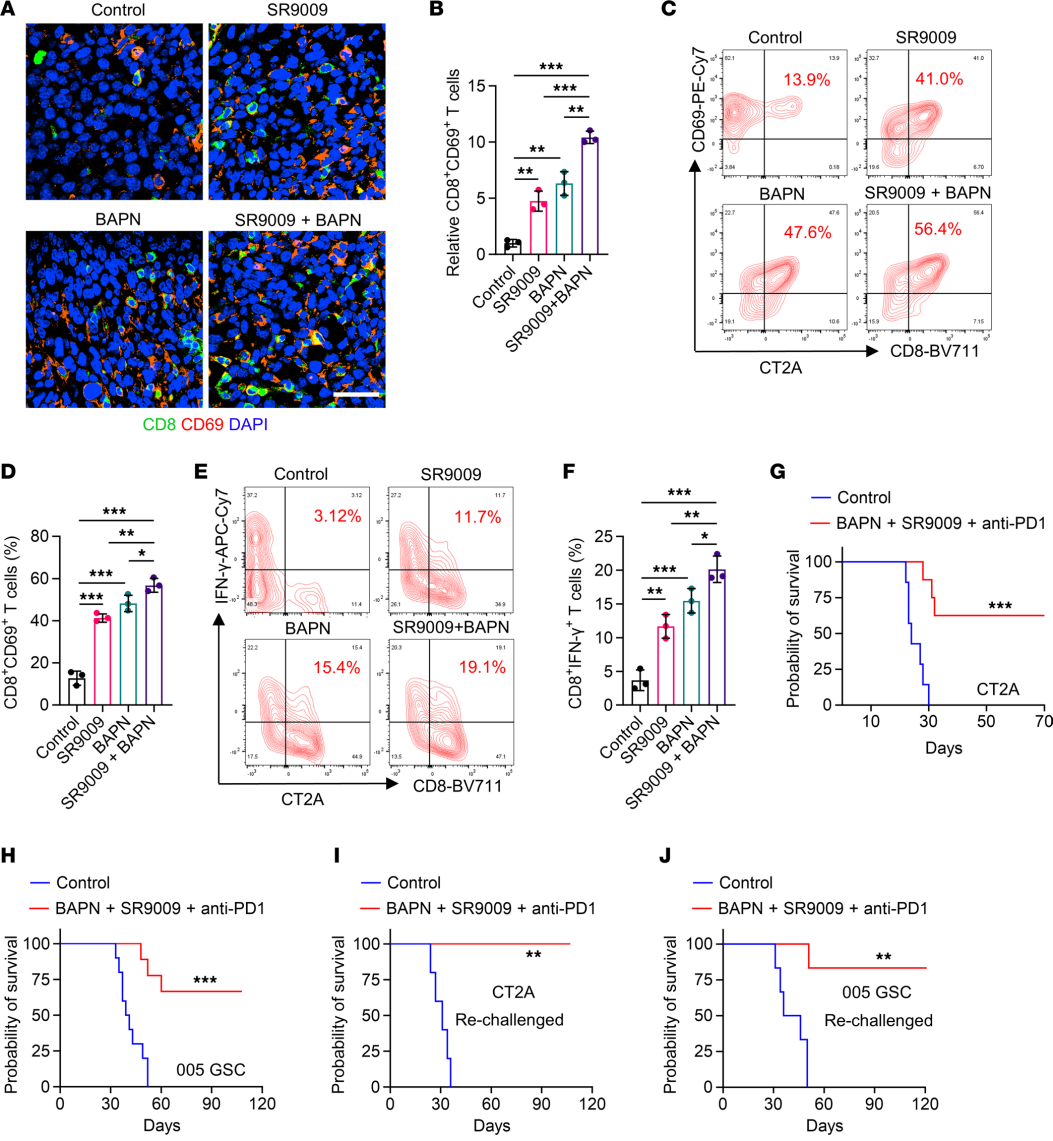
Dual Inhibition of LOX and CLOCK-OLFML3 axis activates antitumor immune response and synergizes with anti-PD1 therapy. (**A** and **B**) Immunofluorescence (**A**) and quantification (**B**) of relative CD8^+^CD69^+^ T cells in tumors from CT2A model (2 × 10^4^ cells/mouse) treated with or without BAPN (2 g/L in drinking water) on day 4, and/or SR9009 (100 mg/kg/day, i.p.) for 10 days beginning at day 7 after orthotopic injection. Scale bar: 50 μm. *n* = 3 independent samples. 1-way ANOVA test. (**C** and **D**) Representative images (**C**) and quantification (**D**) of flow cytometry for the percentage of intratumoral CD8^+^CD69^+^ T cells in size matched tumors from CT2A tumor–bearing mice treated with or without BAPN (2 g/L in drinking water) on day 4, and/or SR9009 (100 mg/kg/day, i.p.) for 10 days beginning at day 7 after orthotopic injection. *n* = 3 independent samples. 1-way ANOVA test. (**E** and **F**) Representative images (**E**) and quantification (**F**) of flow cytometry for the percentage of intratumoral CD8^+^IFN-γ^+^ T cells in size matched tumors from CT2A tumor-bearing mice treated with or without BAPN (2 g/L in drinking water) on day 4, and/or SR9009 (100 mg/kg/day, i.p.) for 10 days beginning at day 7 after orthotopic injection. *n* = 3 independent samples. 1-way ANOVA test. (**G** and **H**) Survival curves of C57BL/6J mice implanted with CT2A cells (2 × 10^4^ cells/mouse, **G**) or 005 GSCs (2 × 10^5^ cells/mouse, **H**). Mice were treated with BAPN (2 g/L in drinking water) on day 4, SR9009 (100 mg/kg/day, i.p.) for 10 days beginning at day 7 after orthotopic injection, and anti-PD1 (10 mg/kg, i.p.) on days 11, 14, and 17. *n* = 7–10 mice per group. Log-rank test. (**I** and **J**) Cured mice from the triple therapy were rechallenged on day 70 with CT2A cells (2 × 10^4^ cells/mouse, **I**) or on day 110 with 005 GSCs (2 × 10^5^ cells/mouse, **J**). Similarly aged naive mice were implanted as controls. *n* = 5 mice per group. Log-rank test. *P < 0.05; **P < 0.01; ***P < 0.001.

Similar to the survival benefits induced by LOX inhibition (Figure 1, K and L), we found that CLOCK inhibition using SR9009 combined with anti-PD1 therapy resulted in survival extension, but did not cure any tumor-bearing mice, in CT2A and 005 GSC models (Supplemental Figure 6, A and B). Given the compensatory upregulation of microglia upon LOX inhibition, we hypothesized that dual targeting macrophages and microglia using BAPN and SR9009 would produce potent antitumor immunity in *PTEN*-deficient GBM. IF staining demonstrated that intratumoral CD8^+^ T cells (Supplemental Figure 6, C and D) and activated CD8^+^ (CD8^+^ CD69^+^) T cells (Figure 6, A and B) were increased upon the treatment with BAPN or SR9009, and these enhancements were further heightened when these 2 treatments were combined. Increases of activated CD8^+^ T cells induced by the treatment with BAPN, SR9009, and their combination were confirmed by flow cytometry for CD45^+^CD3^+^CD8^+^CD69^+^ and CD45^+^CD3^+^CD8^+^IFN-γ^+^ activated T cells in both CT2A and 005 GSC tumors (Figure 6, C–F and Supplemental Figure 6, E–I). In preclinical trials, the triple therapy with BAPN, SR9009, and anti-PD1 resulted in a significant survival extension in both CT2A (Figure 6G) and 005 GSC models (Figure 6H). Notably, 63% and 67% of CT2A and 005 GSC tumor-bearing mice cleared their tumors after the therapy (Figure 6, G and H). The triple therapy (BAPN + SR9009 + anti-PD1) activated T cell memory, as almost all the mice that had previously cleared GBM tumors efficiently suppressed tumor growth when rechallenged with CT2A cells or 005 GSCs and remained tumor free (Figure 6, I and J). Together, these findings suggest that the triple therapy targeting macrophage and microglia infiltration, combined with anti-PD1 therapy, is a promising therapeutic strategy for *PTEN*-deficient GBM.

## Discussion

In this study, we uncover a mechanism underlying the negative correlation between macrophages and microglia in the GBM TME, which provides guidance for designing an effective therapeutic strategy that involves dual targeting macrophages and microglia, and in combination with anti-PD1 immunotherapy. We reveal that LOX inhibition in *PTEN*-deficient GBM upregulates OLFML3 to induce a compensatory increase of microglia infiltration into the GBM TME. Dual inhibition of LOX and CLOCK-OLFML3 axis extends the survival of *PTEN-*deficient GBM-bearing mice and leads to disease eradication in majority of tumor-bearing mice when combined with anti-PD1 therapy.

*PTEN* is a tumor suppressor gene that was originally isolated from a homozygous deletion on chromosome 10q23 of human GBM (37, 38). *PTEN* mutation/depletion is observed in about 30%–40% of GBMs (10), which results in PI3K/AKT pathway activation, contributing to tumor progression and radiotherapy resistance (39). In addition to these cell intrinsic effects, recent studies revealed that *PTEN* loss contributes to the generation of an immunosuppressive GBM TME through a variety of mechanisms. For example, *PTEN* loss in GBM cells leads to immune escape via inducing T cell apoptosis (40) and upregulating PD-L1 expression (41). Moreover, our recent studies revealed that *PTEN* deletion in GBM cells results in upregulation of LOX, which, in turn, triggers the infiltration of macrophages into the GBM TME (20). In this study, we provide further evidence in *PTEN*-deficient GBM mouse models showing that reducing macrophage infiltration via LOX inhibition enhances antitumor T cell immunity and synergizes with anti-PD1 therapy. These in vivo results coupled with the recent findings observed in patients with GBM showing that *PTEN* mutations and macrophage abundance are enriched in anti-PD1 therapy nonresponders compared to responders (28), reinforces the importance of macrophages in regulating anti-PD1 therapy resistance and supports the treatment strategy of combining LOX inhibitors and anti-PD1 therapy specifically in *PTEN*-deficient GBM. It will be important for future studies to define the cut points for *PTEN* deficiency in which these mechanisms are operational to define a companion biomarker for clinical trial.

The robust infiltration of TAMs is one of the key GBM hallmarks (23, 42). Our recent studies have identified PTEN-LOX and CLOCK-OLFML3 axes as the key factors responsible for the infiltration of macrophages and microglia, respectively (13, 20). However, the understanding of the relationship between macrophages and microglia in the GBM TME is limited. Consistent with the recent findings (43), our scRNA-seq analysis in tumors of patients with GBM demonstrated that macrophages are negatively correlated with microglia. In exploring the molecular mechanism underlying this connection, we observed that macrophage chemokine LOX negatively regulates the expression of microglia chemokine OLFML3 in GBM cells by regulating the NF-κB-PATZ1 signaling axis. In vivo, suppressing macrophage infiltration via LOX inhibition induces a compensatory increase of microglia, consistent with findings observed in *Ccr2*-KO GBM tumors (43). These findings encouraged us to explore the possibility of developing a combination therapy of suppressing the infiltration of both macrophages and microglia via inhibition of LOX and CLOCK-OLFML3 axis (13, 20) in *PTEN*-deficient GBM mouse models. This hypothesis is supported by our data showing that dual blockade of macrophage and microglia infiltration using BAPN and SR9009 (13, 20) generates higher antitumor activity relative to monotherapy. Given the known immunosuppressive function of TAMs, increasing evidence shows that depleting and reprogramming TAMs could synergize with ICIs in GBM (23, 25, 27, 35, 42, 44). Previous efforts have centered on developing CSF1R inhibitors to deplete TAMs in GBM, but the results showed that CSF1R inhibition only induces a transient antitumor effect caused by the compensatory changes in macrophages after the treatment in brain tumors (45, 46). Combined anti-CSF1R and anti-PD1 therapies in GBM mouse models shows a modest effect to extend survival (47). Consistent with these preclinical findings, a clinical trial with CSF1R inhibitor showed a minimal antitumor effect in recurrent GBM (48). However, it should be noted that 2 patients with mesenchymal GBM, tumors in which LOX expression and *PTEN* deficiency are high, showed extended progression-free survival in response to CSF1R inhibitor treatment (48). In this study, our findings highlight that dual targeting macrophage and microglia infiltration using BAPN and SR9009 coupled with anti-PD1 therapy produces robust antitumor effect and leads to a sustained long-term antitumor memory response in *PTEN*-deficient GBM mouse models.

In summary, our study not only reveals the molecular mechanism underlying the macrophage-microglia connection in the GBM TEM, but also informs an effective triple therapy for *PTEN*-deficient GBM. However, these findings should be interpreted with caution given our study specifically focuses on *PTEN*-deficient GBM, which only account for 30%–40% of GBM cases. It will be interesting to determine whether the conclusion of this study can be extended to *PTEN*-WT GBM. Moreover, the observed effects of LOX and CLOCK inhibition on immune compartments (e.g., macrophages and microglia) may relate to vascular changes in the GBM TME. Although our previous studies have shown that LOX and CLOCK inhibition reduces tumor angiogenesis in GBM (20, 49), further studies are needed to evaluate whether these treatments affect vascular architecture and vessel leakage in GBM tumors.

## Methods

### Sex as a biological variable.

Sex was not considered as a biological variable in this study. Female C57BL/6J mice were used in this study.

### Cell culture.

The GBM cell lines U87, U251, SF763, and CT2A, as well as 293T cells were cultured in DMEM (Gibco, no. 11995-065). The mouse glioma cell line GL261 cells and SIM-A9 microglia were cultured in DMEM-Ham’s F12 medium (Gibco, no. 10565-018). HMC3 microglia were cultured in Eagle’s Minimum Essential Medium (ATCC, no. 30-2003). THP-1 macrophages were cultured in RPMI 1640 medium (Gibco, no. 11875093). All cell lines were cultured in the indicated medium containing 10% FBS (Thermo Fisher Scientific, no. 16140071) and 1:100 antibiotic-antimycotic (Gibco, no. 15140-122), and were purchased from the American Type Culture Collection (ATCC). Human GSCs (GSC23 and GSC7-10) and mouse GBM tumor-derived 005 GSCs and QPP7 GSCs were cultured in neural stem cell (NSC) proliferation media (Sigma-Aldrich, no. SCM005) containing 20 ng/mL epidermal growth factor (EGF; PeproTech, no. AF-100-15) and basic fibroblast growth factor (bFGF; PeproTech, no. 100-18B). Human GSCs were gifted by Frederick F. Lang from the Brain Tumor Center (The University of Texas MD Anderson Cancer Center). 005 GSCs and QPP7 GSCs were provided by Samuel D. Rabkin (Massachusetts General Hospital, Boston, Massachusetts, USA) and Jian Hu (The University of Texas MD Anderson Cancer Center), respectively. We generated *PTEN* CRISPR KO in SF763 cells as described previously (20). All cells were confirmed to be mycoplasma free and were maintained at 37°C and 5% CO_2_. Cells were treated with BAPN (Sigma-Aldrich, no. B-A3134, 200 μM), SR9009 (Cayman, no. 11929, 5 μM), SC75741 (MedChemExpress, no. HY-10496, 5 μM), and/or NF-κΒ activator 1 (MedChemExpress, no. HY-134476, 1 μM) for 24 hours for protein expression analysis or 8 hours for mRNA expression analysis.

### Mice and intracranial xenograft tumor models.

Female C57BL/6J mice at 3 to 4 weeks of age were purchased from the Jackson Laboratory (no. 0000664). All animals were grouped by 5 mice per cage and maintained in IVC System for a week before the experiment. The intracranial xenograft tumor models were established as described previously (13, 49, 50). In brief, mice were anesthetized by isoflurane through IMPAC6 Anesthesia System. Then a dental drill was used to open a small hole in the skull of mice 1.2 mm anterior and 3.0 mm lateral to the bregma. Mice were placed into the stereotactic apparatus, and 5 μL 005 GSC, CT2A, or GL261 cells in FBS-free culture medium were injected into the right caudate nucleus 3.0 mm below the surface of the brain using a 10 μL Hamilton syringe with an unbeveled 30-gauge needle. The incision was closed using Vetbond glue. Meloxicam (20 mg/kg, daily) was subcutaneously injected for pain relief for 3 days after surgery. Mice were assigned into different groups under blinded conditions after a week of intracranial injection and received treatments with BAPN (2 g/L in drinking water) on day 4, SR9009 (100 mg/kg/day, i.p.) for 10 days beginning at day 7 after orthotopic injection, anti-PD1 (10 mg/kg body weight, i.p.,) on days 11, 14, and 17 after orthotopic injection, and/or clodronate liposomes (200 μL, once every 3 days) starting at day 4 after orthotopic injection. Mice with neurological deficits or moribund appearance were sacrificed according to the IACUC protocol. At the end of the experiment, the brains of mice were collected, either fixed in 4% paraformaldehyde (PFA) (Thermo Fisher Scientific, no. J61899.AK) after transcardiac perfusion with PBS for optimal cutting temperature–cryosectioning (OCT-cryosectioning) or processed using the percoll density gradient cell separation method to isolate tumor-derived immune cells for flow cytometry analysis.

### Mass spectrometry.

A high-performance liquid chromatography tandem mass spectrometry (LC-MS/MS) assay was developed to quantify BAPN and SR9009 in plasma and brains of C57BL/6J mice. Specifically, the blood and brain tissues were collected after 1, 2, 4, and 8 hours of the administration of BAPN (2 g/L in sterile purified water) or SR9009 (100 mg/kg body weight, i.p.). The plasma was generated using the standard centrifugation techniques, and the brain tissues were pulverized by cryogenic grinding with liquid nitrogen. The plasma and brain tissue samples were mixed with internal standards, deproteinized with MeOH, and processed into LC-MS/MS to test the concentration of BAPN or SR9009. The analysis was performed at the Mass Spectrometry Core in Research Resources Center of University of Illinois at Chicago.

### Computational analysis of human GBM datasets.

For analysis of human GBM data, we downloaded the gene expression data of TCGA datasets (Agilent-4502A and/or HG-U133A microarrays) from GlioVis: http://gliovis.bioinfo.cnio.es/ The expression, correlation, and GSEA of interesting genes and gene signatures in patients with GBM were performed.

### GSEA analysis.

GSEA software 4.1.0 (http://www.broad.mit.edu/gsea/software/software_index.html) from the Broad Institute was used. The gene expression data from microarray data of public available GEO and our newly generated RNA sequencing data of U87 cells were used for performing GSEA. The gene Ontology Biological Process (GOBP) signatures were downloaded from the Molecular Signatures Database (51). The normalized enrichment score (NES) and false discovery rate (FDR) were acquired by the analysis, with FDR < 0.25 was considered statistically significant.

### Single-cell sequencing data analysis.

The scRNA-seq data of GEO accession no. GSE131928 (31), were used to analyze expression pattern of *LOX* in glioma cells and the distribution of myeloid cells, including macrophages, monocytes, microglia, and DCs, in tumors of patients with glioma (LGG, ndGBM and rGBM). Based on their abundance in GBM tumors, the correlation between macrophages and microglia was analyzed.

### Plasmids and viral transfections.

For gene knockdown, short hairpin RNA (shRNA) targeting human *LOX* and *PATZ1* and mouse *Clock* in the pLKO.1 vector (Sigma-Aldrich, no. SHC001) were used. Lentiviral particles were generated as we described previously (13, 20). In brief, 8 μg of the shRNA plasmid, 4 μg of the psPAX2 plasmid (Addgene, no. 12260), and 2 μg of the pMD2.G plasmid (Addgene, no. 12259) were transfected into 293T cells plated in 100-mm dishes using Lipofectamine 2000 (Invitrogen, no. 13778150). Supernatant with lentiviral particles was collected and filtered at 48 and 72 hours after transfection. Cells were infected with viral supernatant containing 10 μg/mL polybrene (Millipore, no. TR-1003-G). After 48 hours, cells were selected by puromycin (10 μg/mL; Millipore, no. 540411) and tested for the expression of LOX, PATZ1, and CLOCK by immunoblots. The following human and mouse shRNA sequences (*LOX*: no. 3: TRCN0000286463 and no. 4: TRCN0000286532; *PATZ1*: no. 4: TRCN0000274379 and no. 5: TRCN0000274416; and *Clock*: no. 1: TRCN0000095686 and no. 2: TRCN0000306474) were selected for further use following the validation.

For gene overexpression, plasmids of human Tagged Lenti ORF Clone of *PATZ1* (Origene, no. RC211869L4) and mouse Tagged Lenti ORF Clone of *Lox* (Origene, no. MR206463L4) were used. These plasmids were transformed into high-efficiency chemically competent *Escherichia coli* cells (Thermo Fisher Scientific, no. C737303) and recovered in Lysogenia broth (LB, Thermo Fisher Scientific, no. BP9723). After recovery, LB containing E. coli transformants were plated on LB selection plates containing 34 μg/mL chloramphenicol (Thermo Fisher Scientific, no. BP904) for 16 hours of incubation at 37°C to select clones containing the gene expression vectors. The selected colonies were picked from the selection plates for inoculation in LB broth supplemented with 34 μg/mL chloramphenicol to maintain the selection, and then further purified for plasmid DNA using a QIAprep Spin Miniprep Kit (Qiagen, no. 27106). Purified plasmids were transfected into cells using lentiviral transfection methodology as previously described (13, 20, 25).

### IF.

IF was performed using a standard protocol as previously described (13, 20). In brief, slides from cryosections were kept at room temperature for 30 minutes and fixed in 10% PFA for 30 minutes prior to permeabilization. Then, 0.25% Triton X-100 (Sigma-Aldrich, no. 9036-19-5) in PBS was added for 30 minutes at room temperature to permeabilize the cell membrane. After 3 times PBS washing, sections were blocked by 5% goat serum for 30 minutes. Specimens were incubated with primary antibody or PBS control for 1 hour at room temperature and then overnight at 4°C. Then, the unbound primary antibodies were washed out by 3 times PBS for 3 minutes each and corresponding secondary antibody cocktails were prepared and added to the sections for 1 hour incubation. Cell nuclei were counterstained with DAPI/antifade mounting medium (Vector Laboratories, no. H-1200-10). IF images were captured using Nikon AX/AXR Confocal Microscope System with an apo 60 1.40 Oil 160/0.17 objective in the Center for Advanced Microscopy (CAM) at Northwestern University. For 1 slide, 3–5 fields of images were captured randomly, and the intensity of the protein signal was determined by Image J. The average quantified value of these 3–5 fields was represented as the protein signal intensity of 1 sample and presented as the individual point in the bar graph. The number of replicates for each experiment is indicated in the figure legends. Antibodies specific to CD8 (Invitrogen, no. PA5-81344), CD69 (Santa Cruz Biotechnology, no. sc-373799), CX3CR1 (Invitrogen, no. 702321), F4/80 (Cell Signaling Technology, no. 30325S), OLFML3 (Invitrogen, no. 702321), Ki67 (Cell Signaling Technology, no. 9129S), and CC3 (Cell Signaling Technology, no. 9661S) were used.

### SeqIF multiplexing and microscopy.

Formalin-fixed paraffin-embedded (FFPE) samples from patients with GBM were collected at Northwestern University and pathologically segmented and graded by the study neuropathologist. Automated multiplexed seqIF staining and imaging were performed on these sections using the COMET platform (Lunaphore Technologies). The multiplexed panel comprised 4 antibodies: GFAP (Abcam, no. ab68428), CD31 (Abcam, no. ab182981), P2RY12 (Atlas Antibodies, no. HPA014518), and CD163 (Abcam, no. ab182422). The 4-plex protocol was generated using the COMET Control Software, and reagents were loaded onto the COMET device to perform seqIF. All antibodies were validated using conventional IHC and/or IF staining in conjunction with corresponding fluorophores and DAPI (Thermo Fisher Scientific, no. D21490). For optimal concentration and best signal-to-noise ratio, all antibodies were tested at 3 different dilutions: starting with the manufacturer-recommended dilution (MRD), MRD/2, and MRD/4. Secondary Alexa fluorophore 555 (Invitrogen, no. A32732) and Alexa fluorophore 647 (Invitrogen, no. A32733) were used at 1:200 or 1:400 dilutions, respectively. The optimizations and full runs of the multiplexed panel were executed using the technology integrated in the Lunaphore COMET platform (characterization 2 and 3 protocols, and seqIF protocols, respectively). The seqIF workflow was parallelized on a maximum of 4 slides, with automated cycles of iterative staining of 2 antibodies at a time, followed by imaging and elution of the primary and secondary antibodies, with no sample manipulation during the entire workflow. All reagents were diluted in Multistaining Buffer (BU06, Lunaphore Technologies). The elution step lasted 2 minutes for each cycle and was performed with Elution Buffer (BU07-L, Lunaphore Technologies) at 37°C. Quenching lasted for 30 seconds and was performed with Quenching Buffer (BU08-L, Lunaphore Technologies). Staining was performed with incubation times set at 4 and 2 minutes for primary antibodies and secondary antibodies, respectively. Imaging was performed with an integrated epifluorescent microscope at 20× magnification with Imaging Buffer (BU09, Lunaphore Technologies) and exposure times set for DAPI 80 milliseconds, Cy5 200 milliseconds, TRITC 400 milliseconds. Image registration was performed immediately after concluding the staining and imaging procedures by COMET Control Software. Each seqIF protocol resulted in a multi-layer OME-TIFF file where the imaging outputs from each cycle were stitched and aligned. COMET OME-TIFF files contain a DAPI image, intrinsic tissue autofluorescence in TRITC and Cy5 channels, and a single fluorescent layer per marker. The intrinsic tissue autofluorescence signals were subtracted from the subsequent cycles and the markers were subsequently pseudocolored for visualization of multiplexed staining results in the Viewer from Lunaphore.

### H&E staining.

Staining was performed using the H&E staining kit (Abcam, no. ab245880) according to a standard protocol. In brief, the FFPE sections were baked at 65 °C for 2 hours and then were subjected to xylene and ethanol for deparaffinization and rehydration. After that, the sections were incubated with hematoxylin, Mayer’s (Lillie’s Modification) for 5 minutes, and then incubated with the Bluing Reagent and Eosin Y Solution (Modified Alcoholic) for 15 seconds and 3 minutes, respectively. After washing, slides were dehydrated in 3 changes of absolute alcohol and the images of tissue sections were captured using TissueFAXS in the CAM at Northwestern University.

### RT-qPCR.

Cells were pelleted and RNA was isolated with the RNeasy Mini Kit (Qiagen, no. 74106), as we previously described (13, 20). RNA was quantified by NanoDrop spectrophotometers and then the All-In-1 5× RT MasterMix (Applied Biological Materials, no. G592) was used to reverse-transcribe RNA into cDNA in T100 Thermal Cycler (Bio-Rad). Quantitative real-time PCR *(*RT-qPCR) was performed with the use of SYBR Green PCR Master Mix (Bio-Rad, no. 1725275) in CFX Connect Real-Time PCR Detection System (Bio-Rad). Primers used for RT-qPCR were listed in Supplemental Table 1. The expression of each gene was normalized to that of housekeeping gene *GAPDH*.

### ChIP-PCR.

ChIP-PCR was performed using the commercial Pierce Magnetic CHIP kit (Thermo Fisher Scientific, no. 26157) as we described previously (49). In brief, *PTEN*-KO SF763 cells were cross-linked with 1% PFA for 10 minutes, and then reactions were quenched using the glycine solution for 5 minutes at room temperature. Cells were then lysed with membrane extraction buffer for 10 minutes on ice, and the chromatin fragmentation was generated by Mnase digestion followed by sonication using 3 20-second pulses at 3-watt power. After that, the solubilized chromatin was incubated with PATZ1 (Santa Cruz Biotechnology, no. sc-393223 X) antibody overnight at 4°C followed by 2 hours incubation with CHIP Grade Protein A/G Magnetic Beads with mixing. Immune complexes were then washed with IP Wash Buffer I 3 times and IP Wash Buffer II once. Elution Buffer was added to the sample for elution at 65°C for 30 minutes. Then proteinase K (20 mg/mL) and NaCl (5M) were added for reverse crosslinking at 65°C for 1.5 hours. Eluted DNA was purified using DNA Clean-Up Column and then used to perform PCR. The *OLFML3* primers were designed according to the E-box of the human *OLFML3* gene and were listed in Supplemental Table 1.

### Immunoblotting.

The protein expression of cells was tested by immunoblotting analysis as we described previously (13, 20). In brief, cells were lysed on ice with RIPA lysis buffer (Thermo Fisher Scientific, no. 89900) supplemented with Protease Inhibitor Cocktail (Thermo Fisher Scientific, no. 78429). BCA Protein Assay Kit (Thermo Fisher Scientific, no. PI23225) was used to measure protein concentration. Protein solution was mixed with the LDS sample buffer and heated at 95°C for 10 minutes. After that, protein samples were loaded to SurePAGE gels (GenScript, no. M00653) and then transferred to 0.2 μm nitrocellulose (NC) membrane (Bio-Rad, no. 1620112) using a preprogrammed standard protocol for 30 minutes in the Trans-Blot Turbo system (Bio-Rad). NC membranes were blocked using 5% dry milk in TBST for 1 hour at room temperature and then incubated with primary antibodies (1:1,000 dilution) overnight at 4°C. After washing 3 times, membranes were incubated with HRP-conjugated anti-mouse (Cell Signaling Technology, no. 7076S) or anti-rabbit (Cell Signaling Technology, no. 7074S) secondary antibodies for 2 hours at room temperature. After washing, membranes were incubated with ECL substrate and imaged under ChemiDoc Touch Imaging System (Bio-Rad). Antibodies were purchased from the indicated companies, which include β-actin (Cell Signaling Technology, no. 3700S), LOX (Abcam, no. ab174316), CLOCK (Cell Signaling Technology, no. 5157S), OLFML3 (Abcam, no. ab111712), PD-L1 (Cell Signaling Technology, no. 64988S), P-P65 (Cell Signaling Technology, no. 3033S), P65 (Cell Signaling Technology, no. 8242S), and PATZ1 (Santa Cruz, no. sc-393223 X).

### Migration assay.

HMC3 microglia (5 × 10^4^) were suspended in serum-free culture medium and seeded into 8.0 μm (Corning, no. 3422) inserts. SIM-A9 microglia (1 × 10^5^) and THP-1 macrophages (1 × 10^5^) were suspended in serum-free culture medium and seeded into 5.0 μm (Corning, no. 3421) inserts. The CM from LOX-depleted or inhibited U87 cells, LOX-overexpressed GL261 cells, or SR9009-treated U87 cells were added to the receiver wells, respectively. After 10 hours, migrated cells were fixed with 4% PFA (Thermo Fisher Scientific, no. J61899.AK) for 30 minutes and stained with crystal violet (Sigma-Aldrich, no. C-3886) for another 30 minutes. The membrane inserts were washed with water and imaged under an EVOS microscope. The number of transferred cells was counted using ImageJ.

### Tumor-derived immune cell isolation.

Mice with neurologic deficits or moribund appearance were sacrificed to harvest their brains. Immune cells in the brain tumors were isolated using the percoll density gradient cell separation method as we previously described (35). In brief, after perfusion with PBS, brains were homogenized on ice with precooled 10 mL HBSS. Then cells were spun down at 300*g* for 10 minutes at 4°C, and were resuspended in 30% Percoll (GE Healthcare, no. 17-0891-01). The solution was gently laid on top of the 70% Percoll and centrifuged at 1,200*g* for 30 minutes at 4°C with accelerator 7 and breaker 0. After removing myelin and debris, the interphase was collected and centrifuged at 300*g* for 10 minutes at 4°C. The cell pellet was resuspended for further analysis.

### Flow cytometry.

The single-cell suspensions were incubated with fixable viability dye (Invitrogen, no. 5211229035) on ice for 10 minutes. After washing with FACS buffer (PBS with 1% BSA), cells were incubated with the TruStain FcX (anti-mouse CD16/32) Antibody (BioLegend, no. 103132) and True-Stain Monocyte Blocker (BioLegend, no. 426102) in 5% BSA for 30 minutes on ice to block Fc receptors and nonspecific binding of the cyanine acceptor fluorophores. Different antibody cocktails, including PerCP/Cy5.5 anti-mouse CD45 (BioLegend, no. 103132), AF488 anti-mouse CD3 (BioLegend, no. 100210), BV711 anti-mouse CD8 (BioLegend, no. 100747), PE/Cy7 anti-mouse CD69 (BioLegend, no. 104512), APC/Cy7 anti-mouse IFN-γ (BioLegend, no. 505850), PE/Cy7 anti-mouse/human CD11b (BioLegend, no. 101216), PE anti-mouse CD68 (BD Bioscience, no. 566386), and BV421 anti-mouse CX3CR1 (BD Bioscience, no. 567531) were added to the samples and incubated for 30 minutes on ice. After washing with FACS buffer, cells were incubated with fixation buffer (BioLegend, no. 420801) overnight. Samples were read through the BD FACSymphony or BD LSRFortessa flow cytometer and analyzed in FlowJo v10.8.1.

### CFSE Proliferation assay.

Cell proliferation was assessed using the CellTrace carboxy fluorescein succinimidyl ester (CFSE) Cell Proliferation Kit (Invitrogen, no. C34554). Briefly, 1 × 10^6^ cells were collected and incubated with CFSE working solution (1:1,000) for 20 minutes at 37 °C. The staining was stopped by adding complete cell culture media. After washing, cells were cultured for 3 days with or without the treatment of BAPN (Sigma-Aldrich, no. B-A3134, 200 μM), or SR9009 (Cayman, no. 11929, 5 μM) in the dark and used for flow cytometry analysis. The percentage of CFSE-positive peaks over the undivided peak (generation 0) was analyzed using FlowJo v10.8.1.

### Colony formation assay.

1 × 10^3^ GBM cells were seeded in each well of 6-well plates with or without the treatment of BAPN (Sigma-Aldrich, no. B-A3134, 200 μM), or SR9009 (Cayman, no. 11929, 5 μM). After 7–10 days, cells were fixed and stained with 0.5% crystal violet in 25% methanol for 1 hour. After 3 times washing by PBS, the plates were scanned, and the colony number was counted using ImageJ.

### Patient samples.

Tumor samples from surgically resected IDH-WT GBMs were collected at Northwestern Memorial Hospital. 3 ndGBM patients (no. ITA-13, male, 62-year-old; no. ITA-19, female, 50-year-old; and no. ITA-26, female, 50-year-old) were diagnosed according to the WHO diagnostic criteria. FFPE blocks and slides were prepared and handled by the Northwestern Central Nervous System Tumor Bank.

### Statistics.

Statistical analyses were performed with 1-way ANOVA tests for comparisons among groups and Student *t* tests for comparisons between 2 groups. Data were represented as mean ± SD. Correlation analysis was conducted using the Pearson test to determine the Pearson correlation coefficient (*R* value) and *P* value. The survival analysis for animal models was determined by conducting Log-rank (Mantel-Cox) test. All statistical analyses were performed using GraphPad Prism 10 (GraphPad Software, USA). *P* < 0.05 was considered statistically significant.

### Study approval.

All animal experiments were performed with the approval of the Institutional Animal Care and Use Committee (IACUC) at Northwestern University. The human tissue protocol (STU00214485) was approved by the Institutional Review Board (IRB) at Northwestern University.

### Data availability.

The scRNA-seq data of GBM patient tumors were obtained from the GEO database (GSE131928). The human TCGA GBM data are available at GlioVis: http://gliovis.bioinfo.cnio.es/ The microarray data of SF763 cells with *PTEN*-KO versus WT were obtained from the GEO database (GSE122284). The RNA-seq data of U87 cells with sh*LOX* versus shC are provided in the Supplemental Table 2. Values for all data points in graphs are reported in the Supporting Data Values file.

## Author contributions

YL and JW performed most experiments. HN and ABH performed multiplex immunofluorescence. YL performed single-cell sequencing data analysis. LP and FZ helped with intracranial injection. FK and HA helped with immunoblotting and ChIP-PCR experiments. PC conceived the project. YL and PC wrote the manuscript.

## Supplementary Material

Supplemental data

Unedited blot and gel images

Supplemental table 2

Supporting data values

## Version 1 10/01/2024

In-Press Preview

## Version 2 11/15/2024

Electronic publication
