# Supplementary material for: Dual targeting macrophages and microglia is a therapeutic vulnerability in models of PTEN-deficient glioblastoma
Source: J Clin Invest. 2024 Oct 1;134(22):e178628. doi: 10.1172/JCI178628 (PMC11563674; doi:10.1172/JCI178628)
Supplement: Supplemental data [file jci-134-178628-s217.pdf]

Supplemental Figure 1

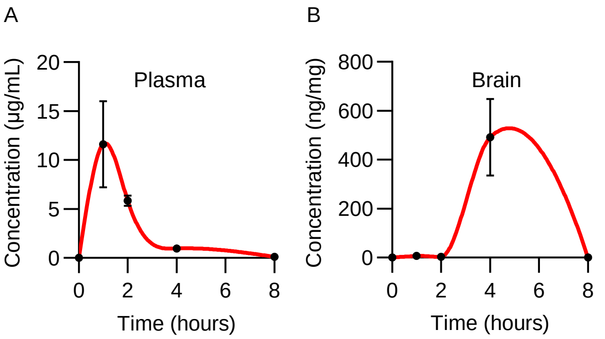

C

Time Post BAPN Administration	Plasma (µg/mL)	Brain (ng/mg)
0h	ND	ND
1h	11.62±4.403	6.561±1.699
2h	5.856±0.536	3.209±0.887
4h	0.965±0.233	491.3±156.8
8h	0.117±0.085	0.671±0.497

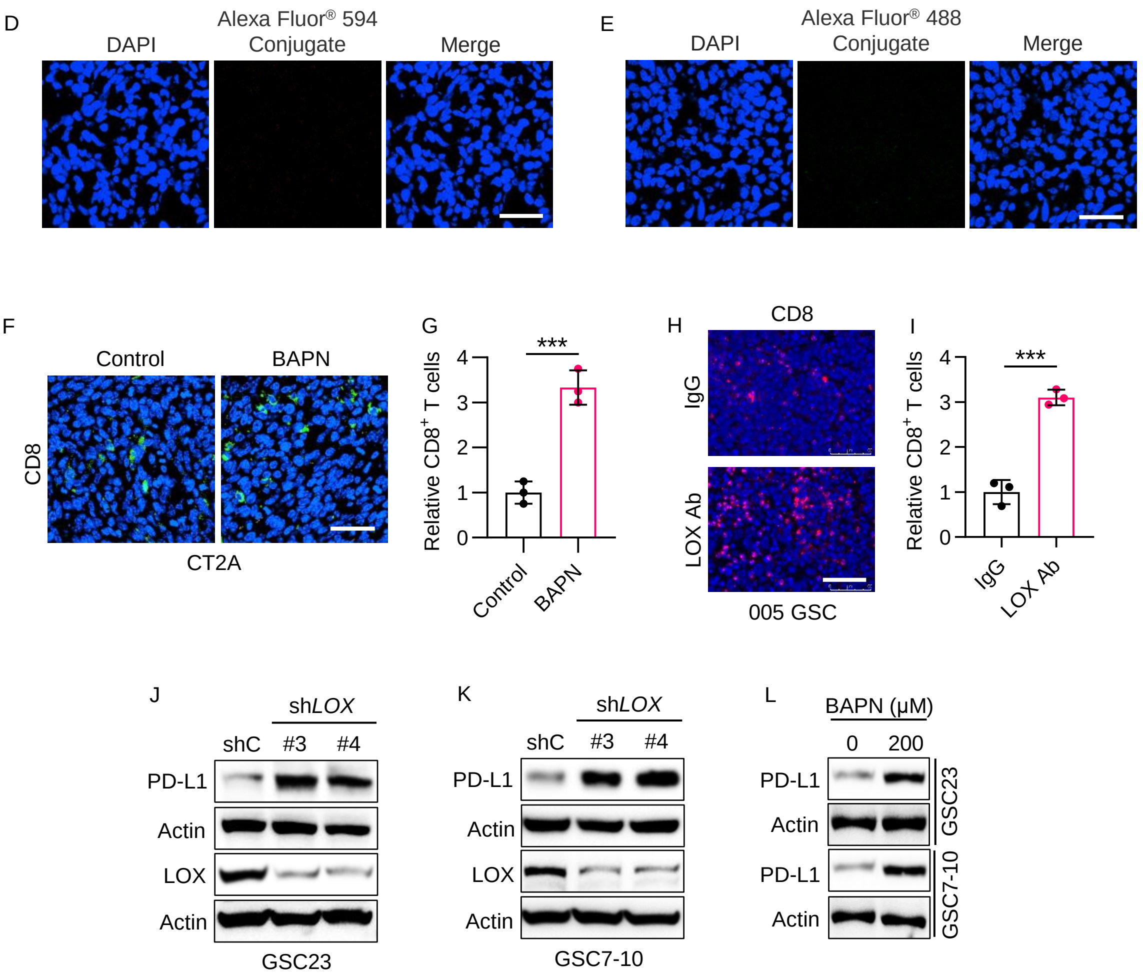

Supplemental Figure 1. Inhibition of LOX increases intratumoral T cell infiltration and upregulates PD-L1 expression in *PTEN*-deficient GBM cells.

(**A-C**) The concentration of BAPN in the plasma (A and C) and brain (B and C) of mice after drug administration at indicated time points. (**D** and **E**) Immunofluorescence images of staining controls using secondary Alexa Fluor® 594 Conjugate (D) and Alexa Fluor® 488 Conjugate antibodies (E). (**F** and **G**) Immunofluorescence (F) and quantification (G) of relative $CD8^+$ T cells in tumors from CT2A model (2×10^4 cells/mouse) treated with or without LOX inhibitor BAPN (2 g/L in drinking water) on day 4. Scale bar, 50 μm. n = 3 independent samples. Student's t test. (**H** and **I**) Immunofluorescence (H) and quantification (I) of relative $CD8^+$ T cells in tumors from 005 GSC-bearing mice treated with IgG or LOX neutralizing antibodies (Ab, 20 mg/kg, i.p., once every 4 days) starting at 4 days post-orthotopic injection. Scale bar, 100 μm. n = 3 independent samples. Student's t test. (**J** and **K**) Immunoblots for PD-L1 and LOX in lysates of GSC23 (J) and GSC7-10 (K) expressing shRNA control (shC) and *LOX* shRNAs (sh*LOX*). (**L**) Immunoblots for PD-L1 in lysates of GSC23 and GSC7-10 treated with BAPN at indicated concentrations. ***, $P < 0.001$.

Supplemental Figure 2

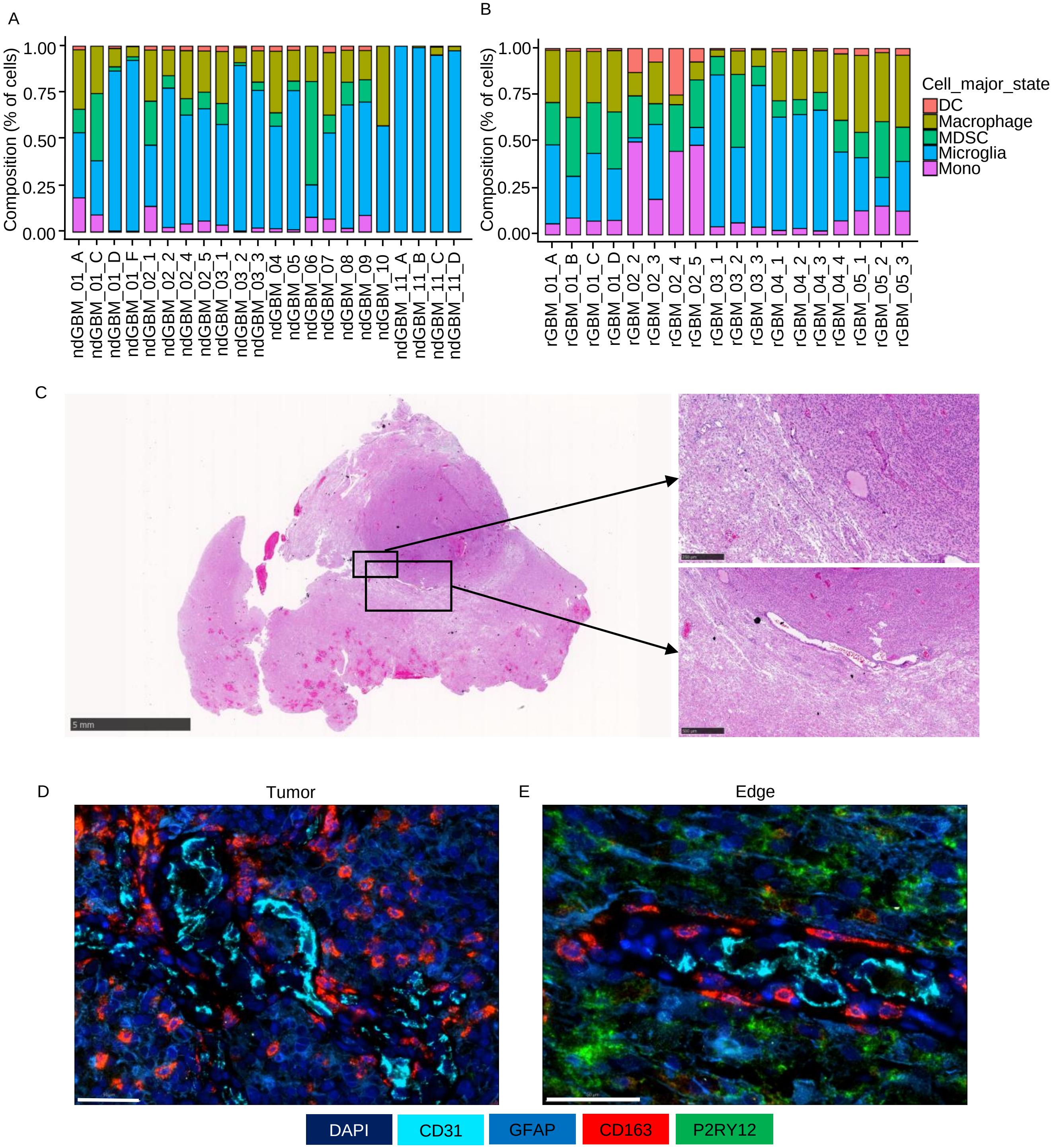

Supplemental Figure 2. Relationship between macrophages and microglia in the GBM TME. (**A** and **B**) Percentage of different myeloid cell cells in tumors of newly diagnosed GBM (ndGBM, A) and recurrent GBM (rGBM, B) from the single cell RNA-sequencing dataset (GSE182109). (**C**) Representative images of H&E staining to designate the tumor edges from IDH1-WT GBM patients. Scale bar, 5 mm (left), 250 μm (right upper), and 500 μm (right bottom). (**D** and **E**) Higher magnified view of $CD31^+$ blood vessels, $P2RY12^+$ microglia and $CD163^+$ macrophages in tumors (D) and tumor edges (E) from IDH1-WT GBM patients. Scale bar, 50 μm.

Supplemental Figure 3

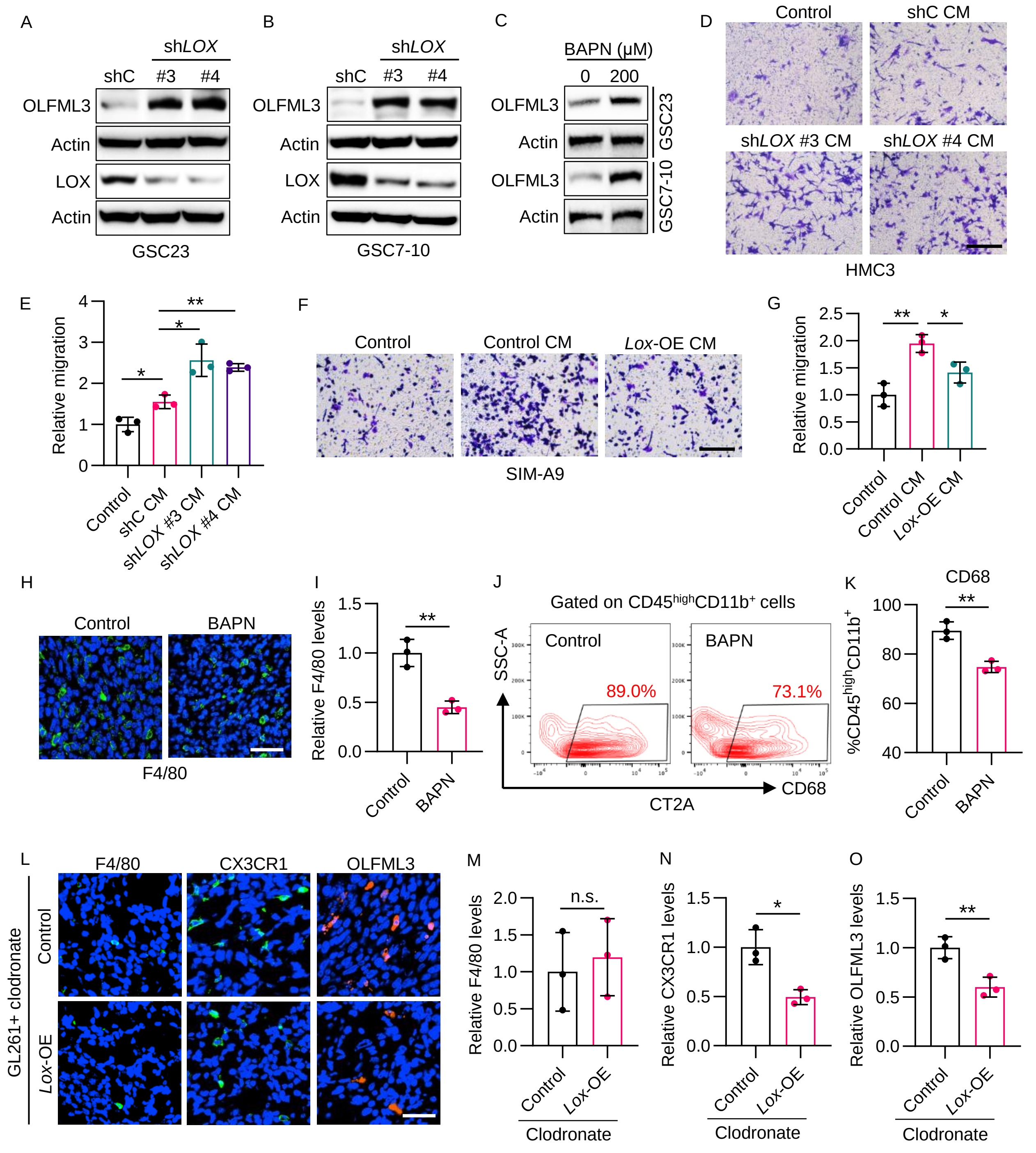

Supplemental Figure 3. LOX is negatively related to OLFML3 expression and microglia infiltration in GBM.

(**A** and **B**) Immunoblots for OLFML3 and LOX in lysates of GSC23 (A) and GSC7-10 (B) expressing shRNA control (shC) and *LOX* shRNAs (sh*LOX*). (**C**) Immunoblots for OLFML3 in lysates of GSC23 and GSC7-10 treated with BAPN at indicated concentrations. (**D** and **E**) Representative images (D) and quantification (E) of relative migration of HMC3 microglia following stimulation with the conditioned media (CM) from U87 cells expressing shC and sh*LOX*. Scale bar, 400 μm. n = 3 independent samples. One-way ANOVA test. (**F** and **G**) Representative images (F) and quantification (G) of relative migration of SIM-A9 microglia following stimulation with the CM from control and LOX-overexpressed GL261 cells. Scale bar, 200 μm. n = 3 independent samples. One-way ANOVA test. (**H** and **I**) Immunofluorescence (H) and quantification (I) of relative F4/80$^+$ macrophages in tumors from CT2A-bearing mice treated with or without BAPN (2 g/L in drinking water) on day 4. Scale bar, 50 μm. n = 3 independent samples. Student's t test. (**J** and **K**) Representative images (J) and quantification (K) of flow cytometry for the percentage of intratumoral CD45^{high}CD11b$^+$CD68$^+$ macrophages in tumors from CT2A-bearing mice treated with or without BAPN on day 4. n = 3 independent samples. Student's t test. (**L-O**) Immunofluorescence (L) and quantification of relative F4/80$^+$ macrophages (M), CX3CR1$^+$ microglia (N), and OLFML3$^+$ cells (O) in tumors from mice implanted with control and LOX-overexpressed GL261 cells and treated with clodronate liposomes (200 μL, once every 3 days) starting at day 4. Scale bar, 50 μm. n = 3 independent samples. Student's t test. *, $P < 0.05$; **, $P < 0.01$; and n.s., not significant ($P > 0.05$).

Supplemental Figure 4

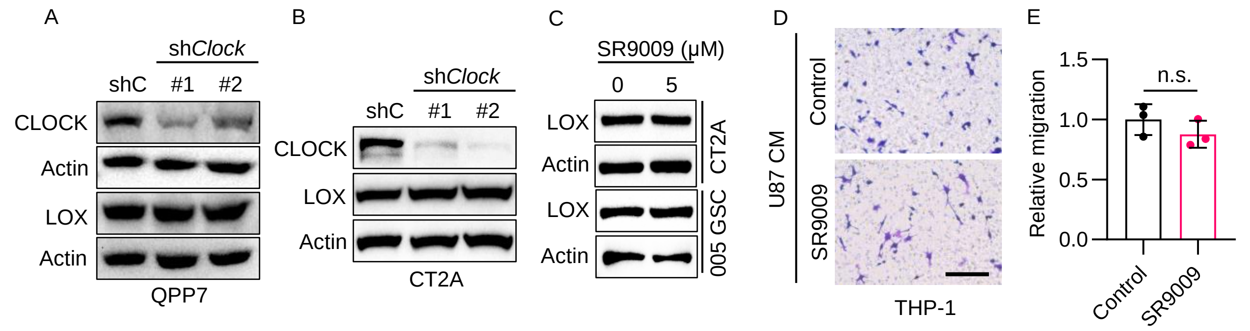

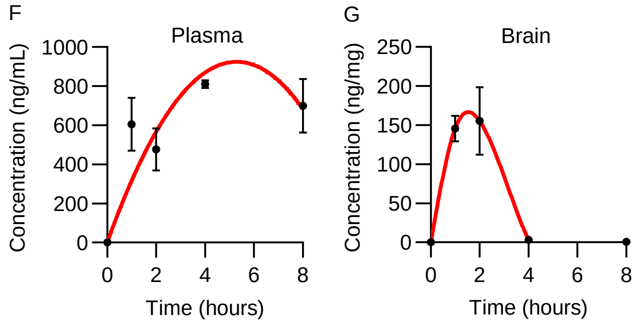

H

Time Post SR9009 Injection	Plasma (ng/mL)	Brain (ng/mg)
0h	ND	ND
1h	605.0±134.8	145.7±16.51
2h	476.7±107.2	155.3±43.26
4h	810.3±19.32	2.874±0.802
8h	699.0±138.0	0.809±0.060

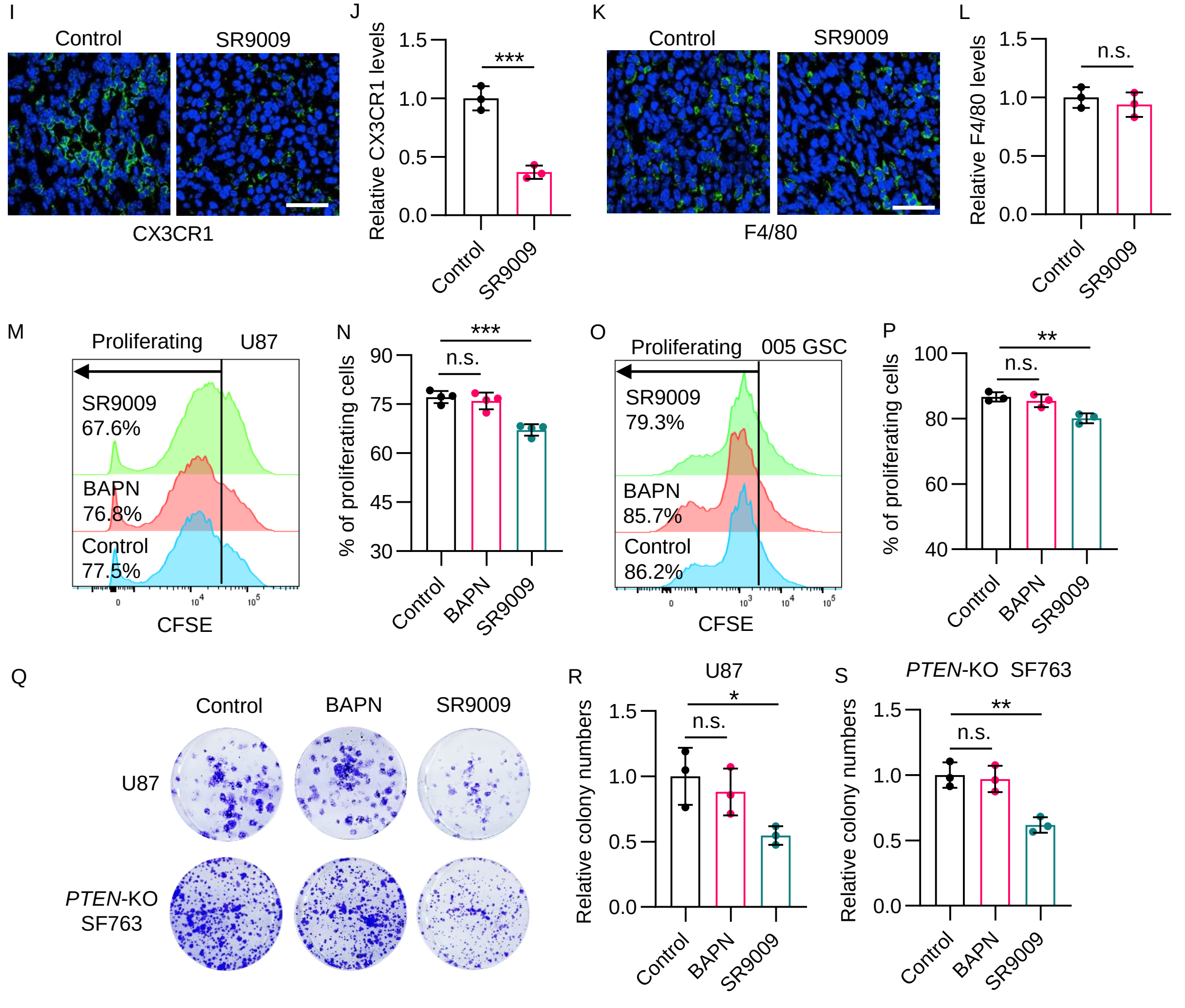

Supplemental Figure 4. CLOCK inhibition did not affect LOX expression and macrophage infiltration in GBM.

(**A** and **B**) Immunoblots for CLOCK and LOX in lysates of QPP7 GSCs (A) and CT2A cells (B) expressing shRNA control (shC) and *Clock* shRNAs (sh*Clock*). (**C**) Immunoblots for LOX in lysates of CT2A cells and 005 GSCs treated with SR9009 (5 μM). (**D** and **E**) Representative images (D) and quantification (E) of relative migration of THP-1 macrophages following stimulation with the conditioned media (CM) from U87 cells pretreated with or without SR9009 (5 μM). Scale bar, 200 μm. n = 3 independent samples. Student's t test. (**F-H**) The concentration of SR9009 in the plasma (F and H) and brain (G and H) of mice after drug administration at indicated time points. (**I** and **J**) Immunofluorescence (I) and quantification (J) of relative $CX3CR1^+$ microglia in tumors from CT2A-bearing mice treated with or without SR9009 (100 mg/kg/day, i.p.) for 10 days beginning at day 7 post-orthotopic injection. Scale bar, 50 μm. n = 3 independent samples. Student's t test. (**K** and **L**) Immunofluorescence (K) and quantification (L) of relative $F4/80^+$ macrophages in tumors from CT2A-bearing mice treated with or without SR9009 (100 mg/kg/day, i.p.) for 10 days beginning at day 7 post-orthotopic injection. Scale bar, 50 μm. n = 3 independent samples. Student's t test. (**M** and **N**) Representative images (M) and quantification (N) of proliferation of U87 cells treated with or without BAPN (200 μM) or SR9009 (5 μM). n = 4 independent samples. One-way ANOVA test. (**O** and **P**) Representative images (O) and quantification (P) of proliferation of 005 GSCs treated with or without BAPN (200 μM) or SR9009 (5 μM). n = 3 independent samples. One-way ANOVA test. (**Q-S**) Representative images (Q) and quantification (R and S) of colony formation of U87 and *PTEN*-KO SF763 cells treated with or without BAPN (200 μM) or SR9009 (5 μM). n = 3 independent samples. One-way ANOVA test. *, $P < 0.05$; **, $P < 0.01$; ***, $P < 0.001$; and n.s., not significant ($P > 0.05$).

Supplemental Figure 5

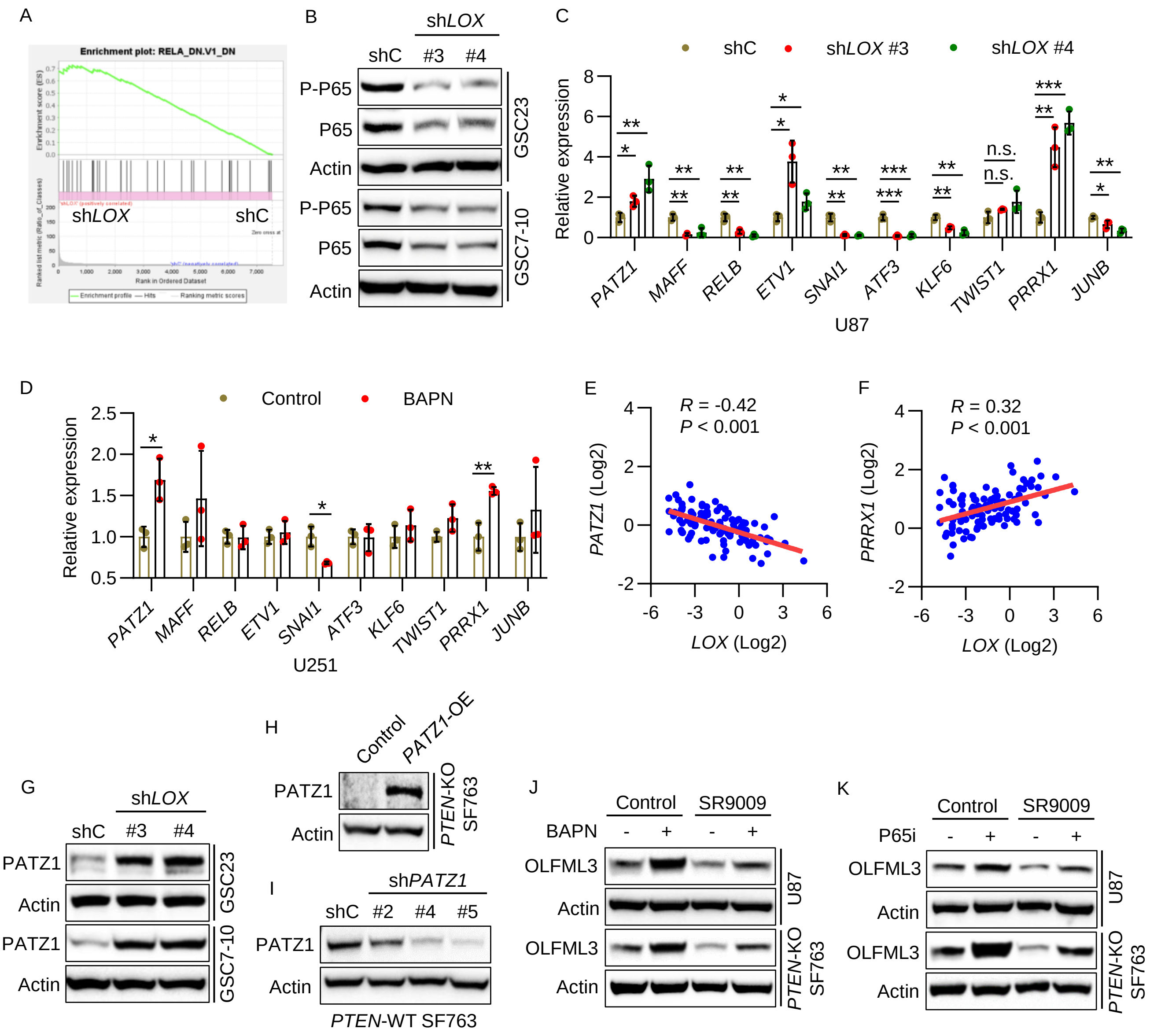

Supplemental Figure 5. LOX regulates the NF-kB-PATZ1 signaling axis independently of CLOCK.

(**A**) Gene Set Enrichment Analysis (GSEA) shows enrichment of RELA_DN.v1_DN signature in *LOX* shRNA (sh*LOX*) versus shRNA control (shC) U87 cells. (**B**) Immunoblots for P-P65 and P65 in lysates of GSC23 and GSC7-10 expressing shC and sh*LOX*. (**C**) Relative mRNA expression of 10 transcription factors (TFs) in U87 cells expressing shC and sh*LOX*. n = 3 independent samples. One-way ANOVA test. (**D**) Relative mRNA expression of 10 TFs in U251 cells treated with or without LOX inhibitor BAPN (200 μM). n = 3 independent samples. Student's t test. (**E**) Relationship between *PATZ1* and *LOX* expression in TCGA GBM patient tumors. Pearson test. (**F**) Relationship between *PRRX1* and *LOX* expression in TCGA GBM patient tumors. Pearson test. (**G**) Immunoblots for PATZ1 in lysates of GSC23 and GSC7-10 expressing shC and sh*LOX*. (**H**) Immunoblots for PATZ1 in lysates of *PTEN*-KO SF763 cells in the presence or absence of PATZ1 overexpression (OE). (**I**) Immunoblots for PATZ1 in lysates of *PTEN*-WT SF763 cells expressing shC and *PATZ1* shRNA (sh*PATZ1*). (**J**) Immunoblots for OLFML3 in lysates of U87 and *PTEN*-KO SF763 cells treated with or without SR9009 (5 μM) or BAPN (200 μM). (**K**) Immunoblots for OLFML3 in lysates of U87 and *PTEN*-KO SF763 cells treated with or without SR9009 (5 μM) or P65 inhibitor (P65i) SC75741 (5 μM). *, $P < 0.05$; **, $P < 0.01$; ***, $P < 0.001$; and n.s., not significant ($P > 0.05$).

Supplemental Figure 6

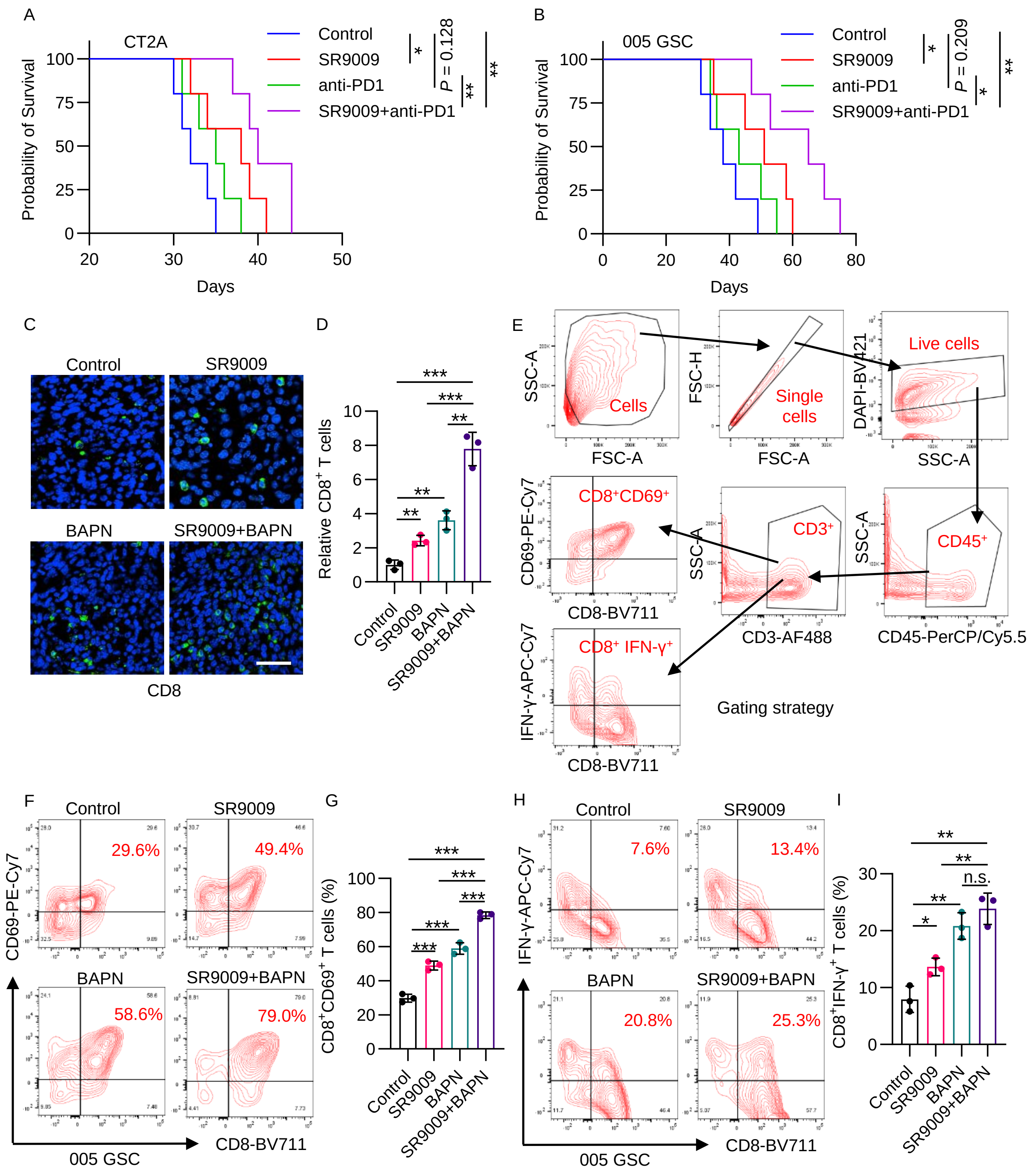

Supplemental Figure 6. Inhibiting LOX and CLOCK-OLFML3 axis activates anti-tumor immune response.

(**A** and **B**) Survival curves of C57BL/6 mice implanted with CT2A cells (2×10^4 cells/mouse, A) or 005 GSCs (2×10^5 cells/mouse, B). Mice were treated with SR9009 (100 mg/kg/day, i.p.) for 10 days beginning at day 7 post-orthotopic injection and then received the treatment with IgG or anti-PD1 (10 mg/kg, i.p.) on days 11, 14, and 17. n = 5 mice per group. Log-rank test. (**C** and **D**) Immunofluorescence (C) and quantification (D) of relative $CD8^+$ T cells in tumors from CT2A model (2×10^4 cells/mouse) treated with or without BAPN (2 g/L in drinking water) on day 4, and/or SR9009 (100 mg/kg/day, i.p.) for 10 days beginning at day 7 post-orthotopic injection. Scale bar, 50 μm. n = 3 independent samples. One-way ANOVA test. (**E**) Gating strategy for flow cytometry of cells from brain tumor tissues. (**F** and **G**) Representative images (F) and quantification (G) of flow cytometry for the percentage of intratumoral $CD8^+CD69^+$ T cells in size matched tumors from 005GSC tumor-bearing mice treated with or without BAPN (2 g/L in drinking water) on day 4, and/or SR9009 (100 mg/kg/day, i.p.) for 10 days beginning at day 7 post-orthotopic injection. n = 3 independent samples. One-way ANOVA test. (**H** and **I**) Representative images (H) and quantification (I) of flow cytometry for the percentage of intratumoral $CD8^+IFN\text{-}\gamma^+$ T cells in size matched tumors from 005GSC tumor-bearing mice treated with or without BAPN (2 g/L in drinking water) on day 4, and/or SR9009 (100 mg/kg/day, i.p.) for 10 days beginning at day 7 post-orthotopic injection. n = 3 independent samples. One-way ANOVA test. *, $P < 0.05$; **, $P < 0.01$; ***, $P < 0.001$; and n.s., not significant ($P > 0.05$).

Supplemental Table 1. A list of primers used for RT-qPCR and ChIP-qPCR.

**Gene name**	**Forward**	**Reverse**
OLFML3	TCCTTTTGTCATGGTCGGGAC	TAAAGCAGCTAGTCGGCGTTC
PATZ1	GATGCACACTATCAGCTCCAAG	CGATAACCGACCTCATCAGCA
MAFF	TGCCCAGGTCCCATTTCTC	GGCCCACGAAGGGAATGT
RELB	CAGCCTCGTGGGGAAAGAC	GCCCAGGTTGTTAAAACTGTGC
ETV1	GGCCCCAGGCAGTTTTATGAT	GATCCTCGCCGTTGGTATGT
SNAI1	TCGGAAGCCTAACTACAGCGA	AGATGAGCATTGGCAGCGAG
ATF3	CCTCTGCGCTGGAATCAGTC	TTCTTTCTCGTCGCCTCTTTTT
KLF6	GGCAACAGACCTGCCTAGAG	CTCCCGAGCCAGAATGATTTT
TWIST1	GTCCGCAGTCTTACGAGGAG	GCTTGAGGGTCTGAATCTTGCT
PRRX1	CAGGCGGATGAGAACGTGG	AAAAGCATCAGGATAGTGTGTCC
JUNB	ACGACTCATACACAGCTACGG	GCTCGGTTTCAGGAGTTTGTAGT
GAPDH	GGAGCGAGATCCCTCCAAAAT	GGCTGTTGTCATACTTCTCATGG
OLFML3 #1-1 (ChIP)	GAGGTGGTTAAGGCTTCTG	CTCTCACTACTCTGGAATGC
OLFML3 #1-2 (ChIP)	GAGGTGGTTAAGGCTTCTG	CTCACTACTCTGGAATGCTT
OLFML3 #2-1 (ChIP)	CCTGCTTGACTGCCTAGA	AGCCAGAAGCCTTAACCA
OLFML3 #2-2 (ChIP)	CCTGCTTGACTGCCTAGA	GCAGCCAGAAGCCTTAAC
OLFML3 #3-1 (ChIP)	GGTGAAGTCTAAGCCTTAGT	GGTCAAGCCCAACTTATTC
OLFML3 #3-2 (ChIP)	GGTGAAGTCTAAGCCTTAGT	AAGGGAGGAAGCAGGAAA
