# Supplementary material for: Dual targeting macrophages and microglia is a therapeutic vulnerability in models of PTEN-deficient glioblastoma
Source: J Clin Invest. 2024 Oct 1;134(22):e178628. doi: 10.1172/JCI178628 (PMC11563674; doi:10.1172/JCI178628)

Full unedited blot for Figure 1

Figure 1G

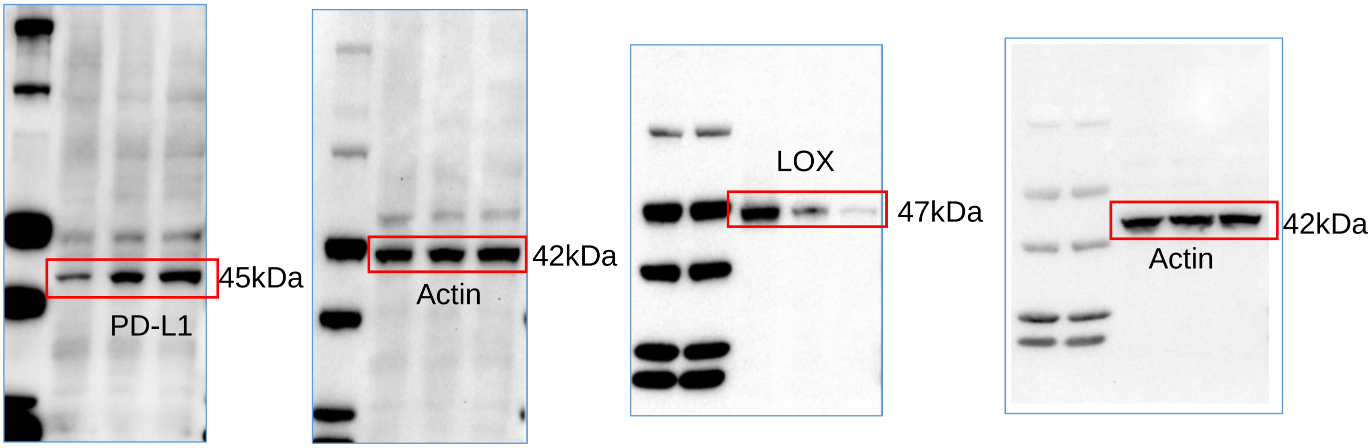

Figure 1H

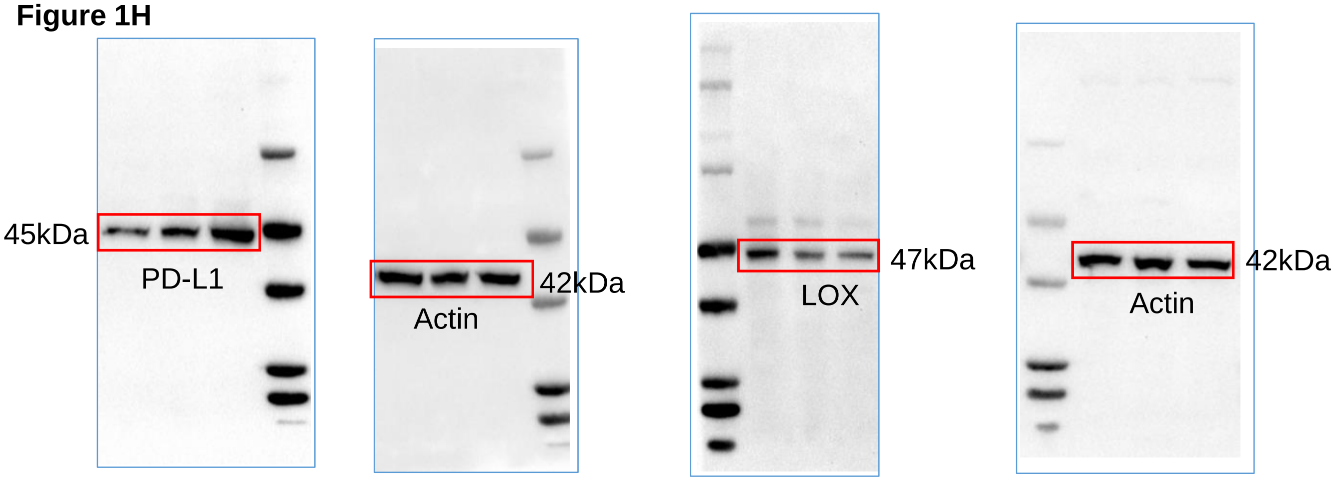

Figure 1I

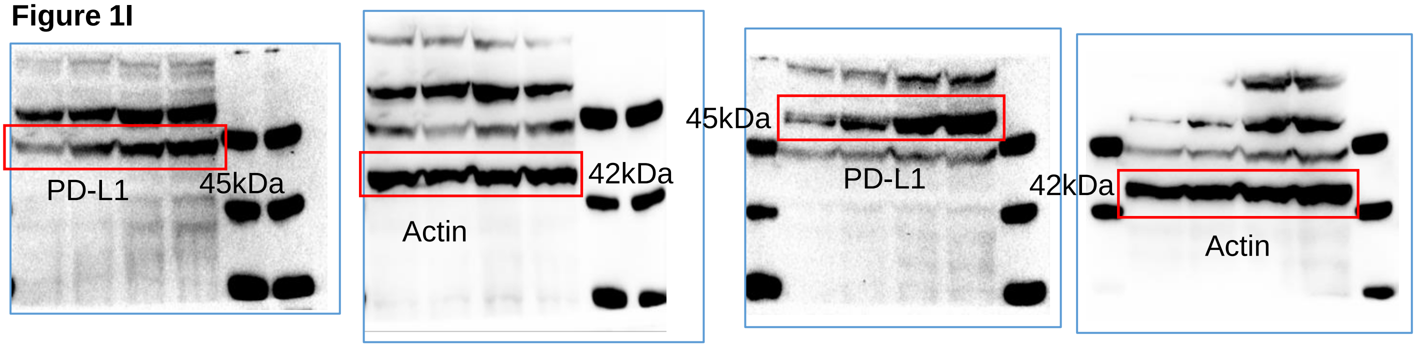

Figure 1J

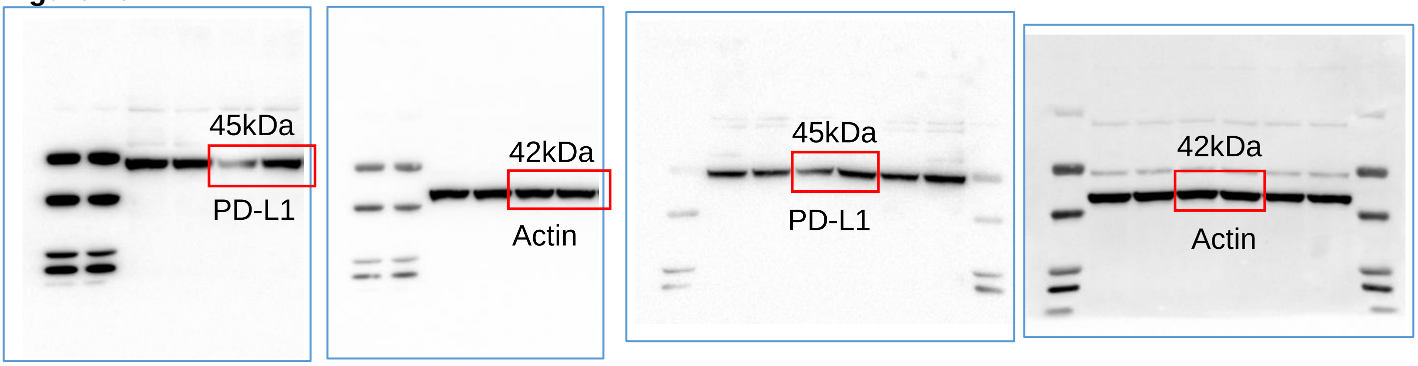

Full unedited blot for Figure 3

Figure 3C

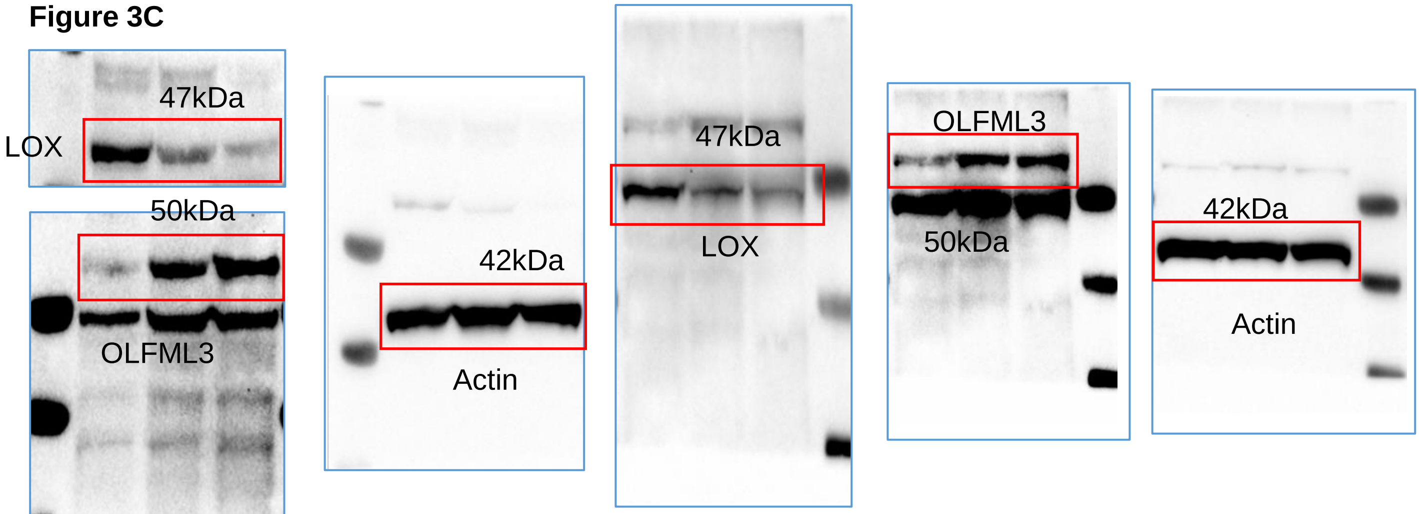

Figure 3D

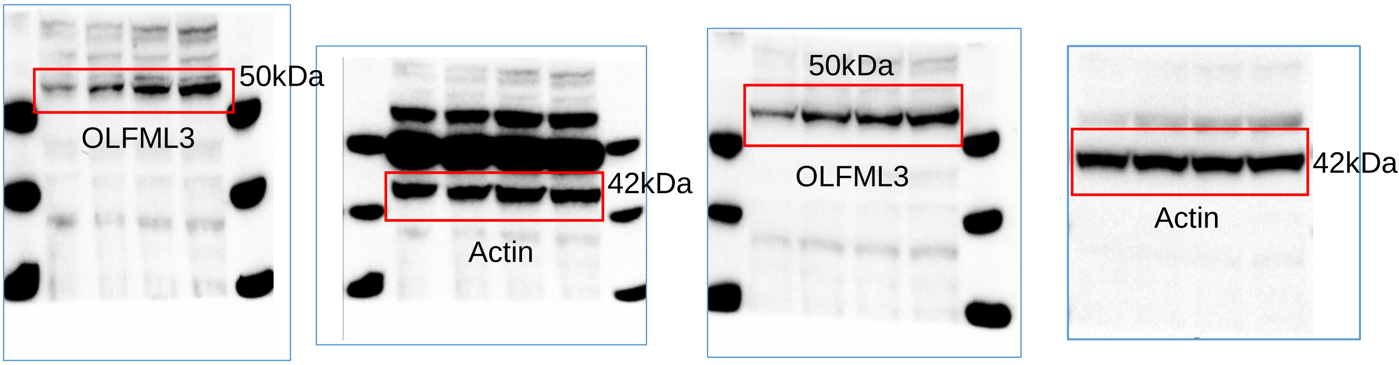

Figure 3E

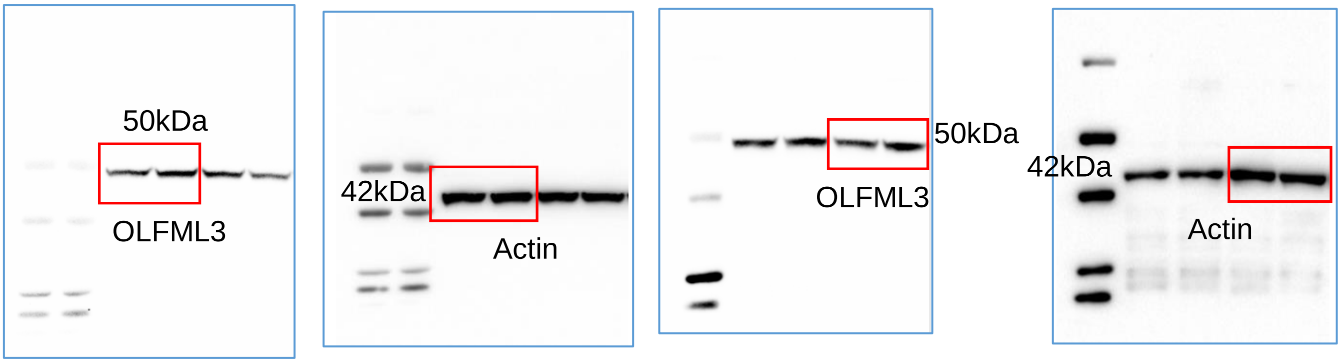

Figure 3H

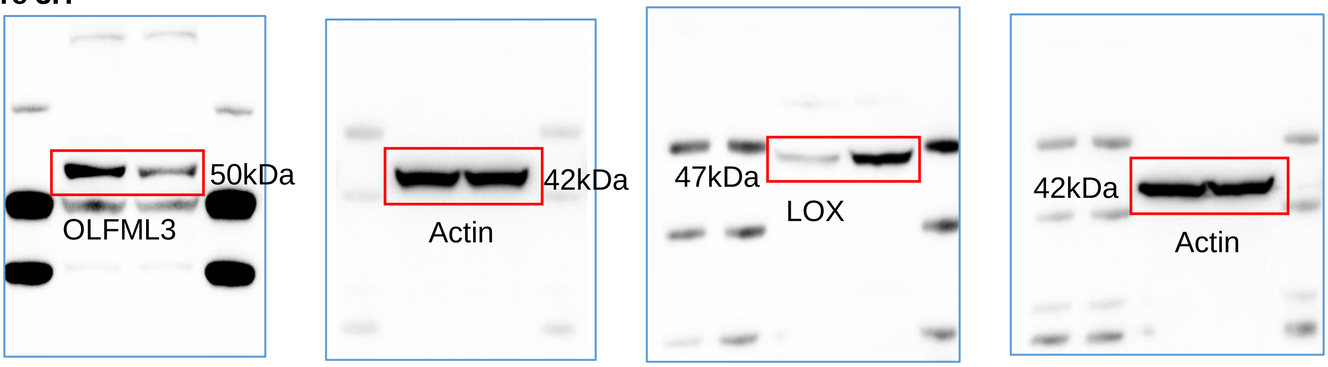

Full unedited blot for Figure 5

Figure 5B

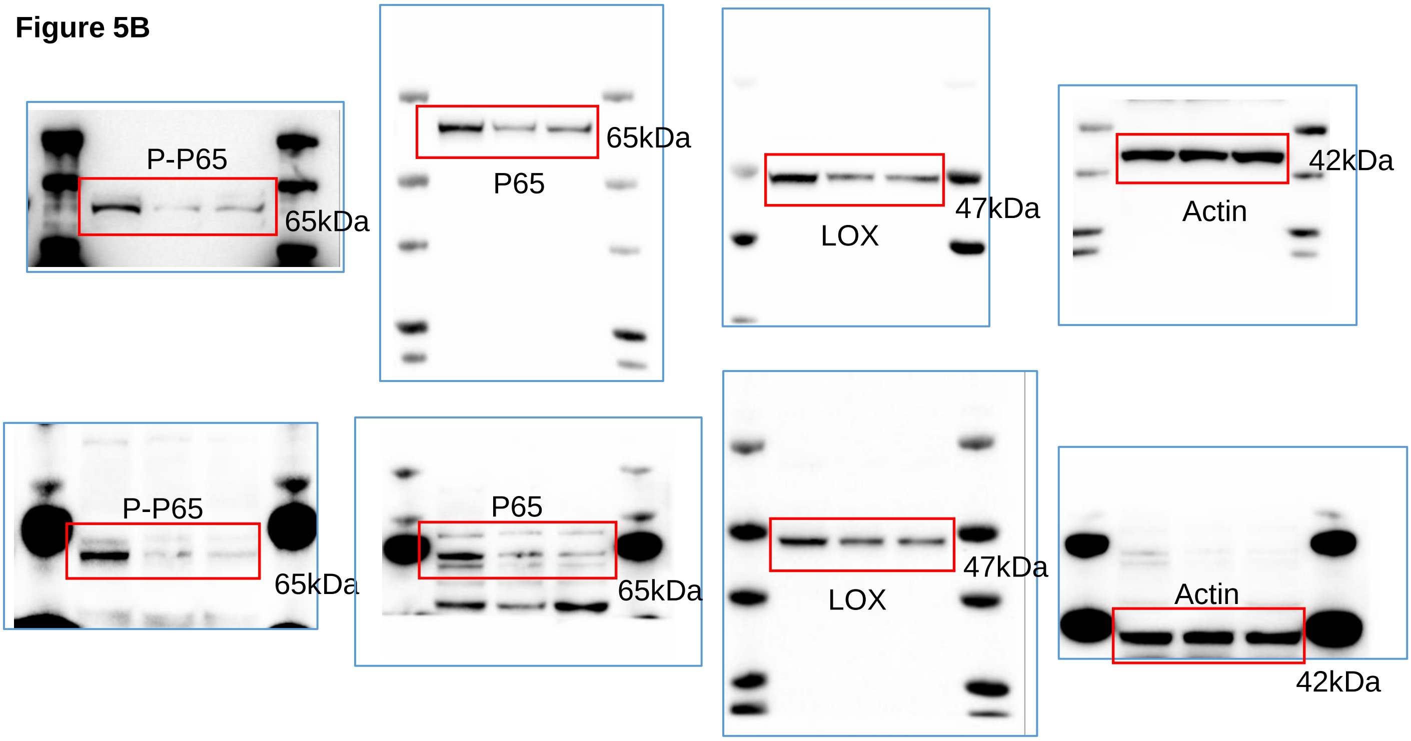

Figure 5G

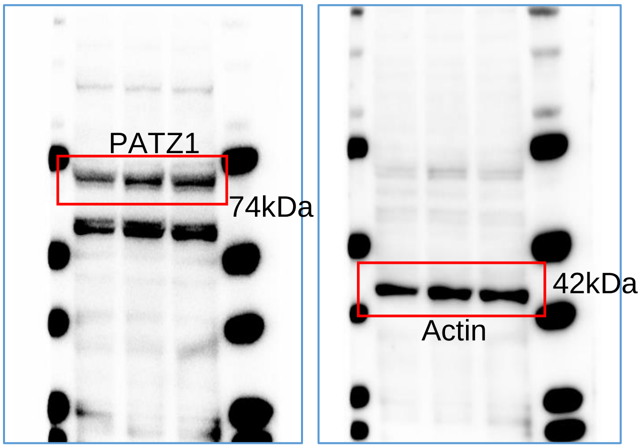

Figure 5K

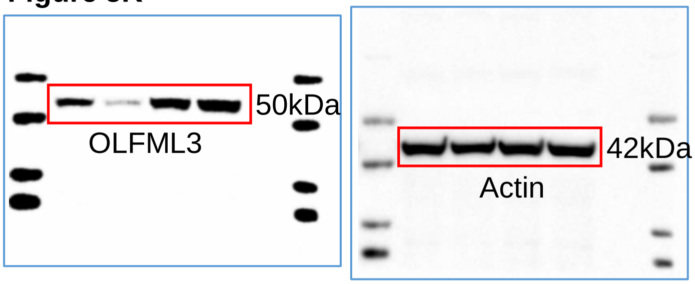

Figure 5L

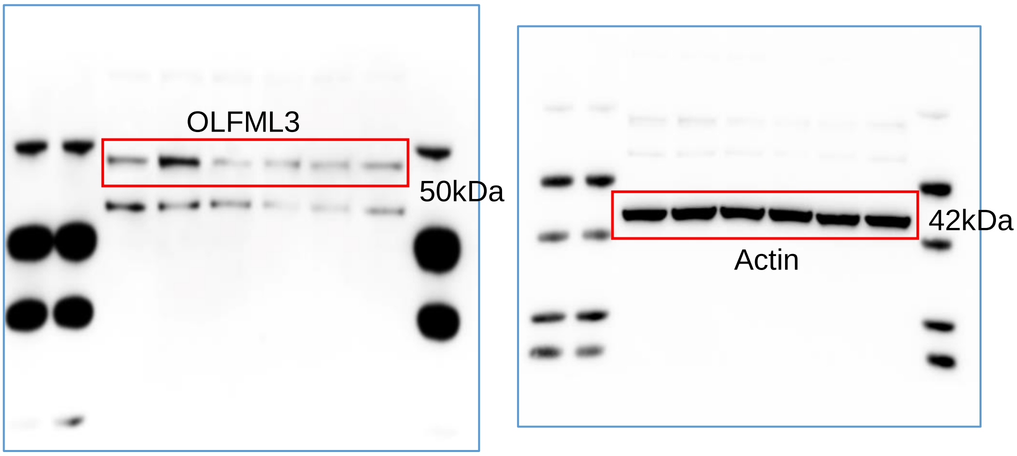

Full unedited blot for Figure S1

Figure S1J

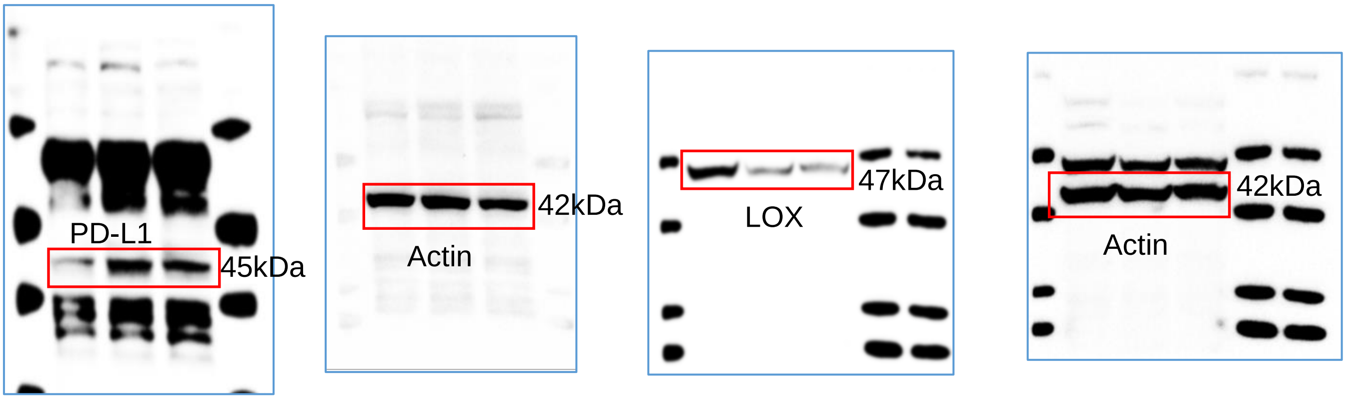

Figure S1K

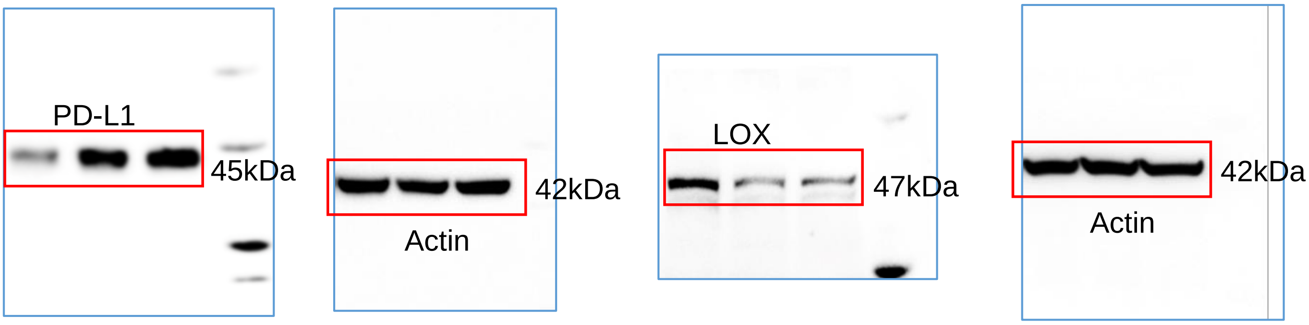

Figure S1L

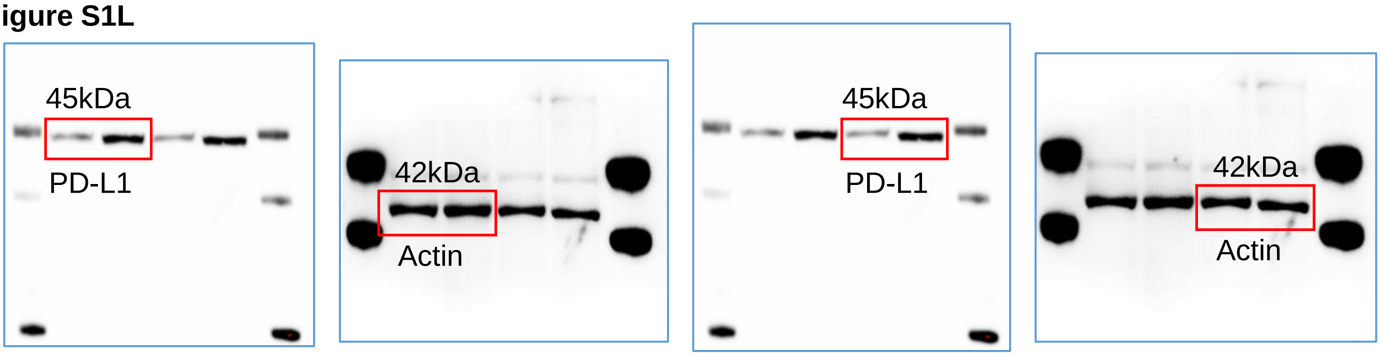

Full unedited blot for Figure S3

Figure S3A

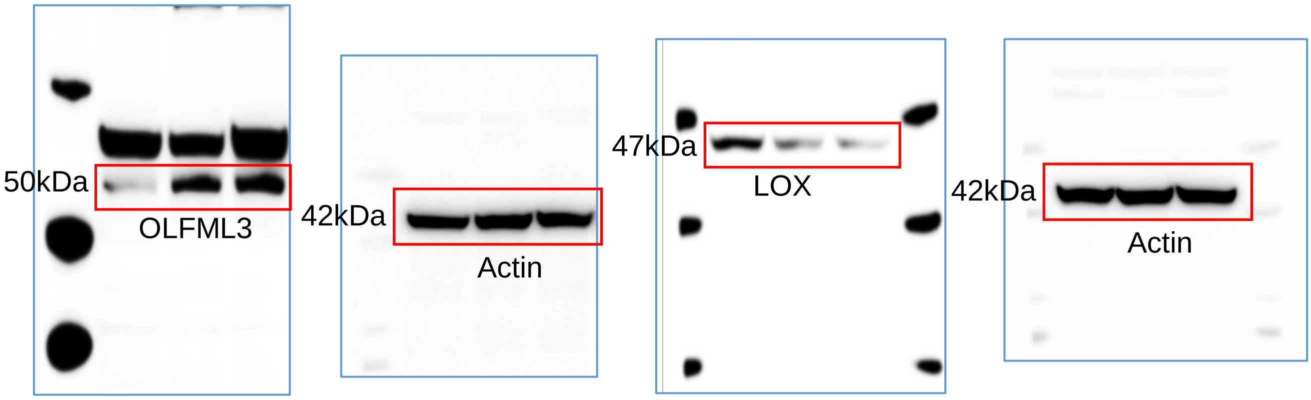

Figure S3B

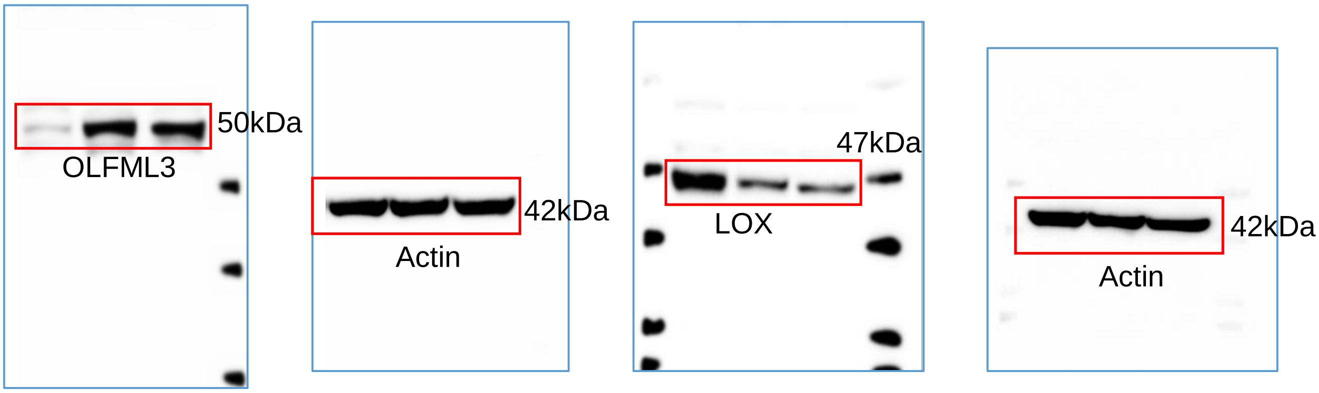

Figure S3C

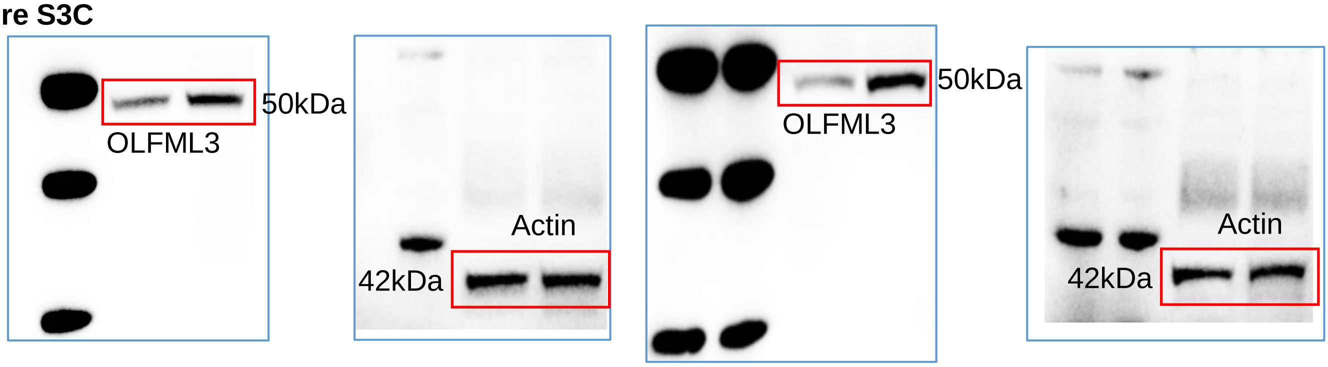

Full unedited blot for Figure S4

Figure S4A

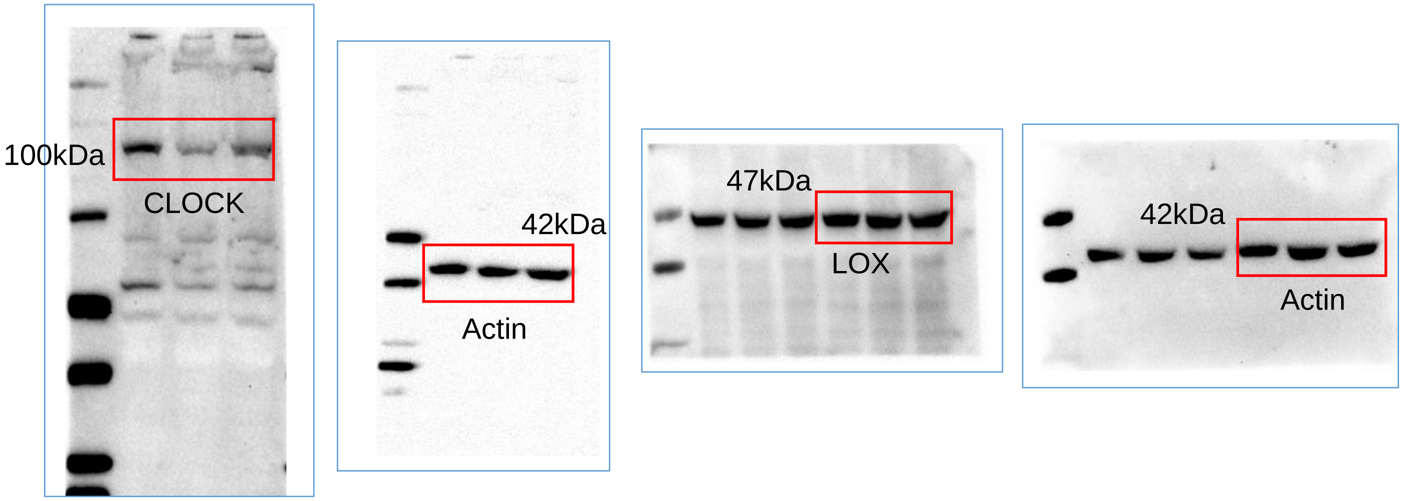

Figure S4B

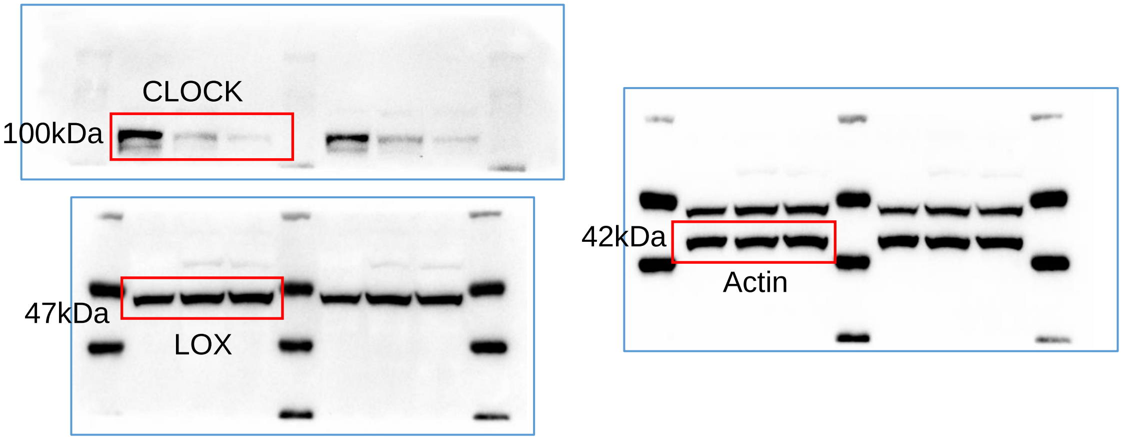

Figure S4C

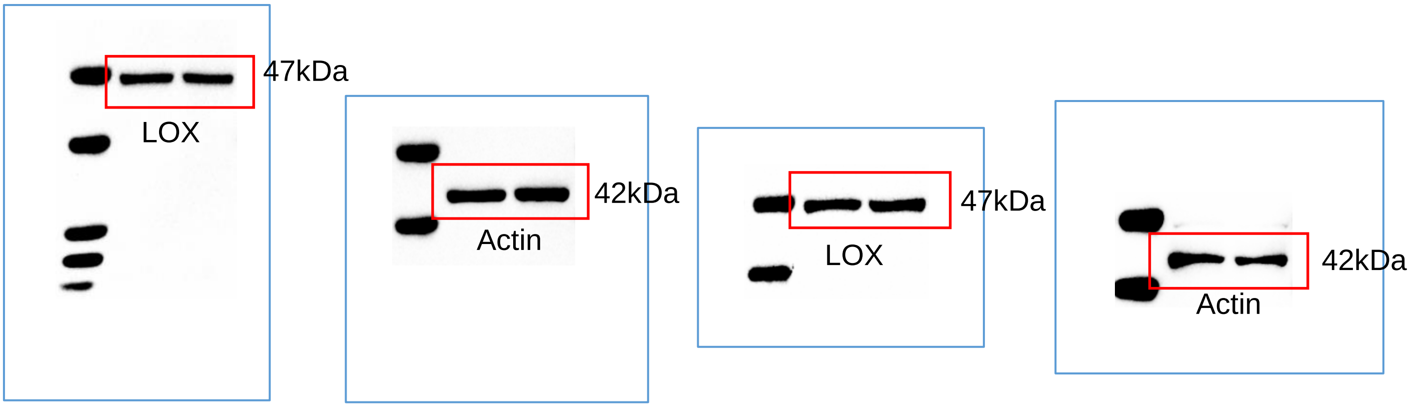

Full unedited blot for Figure S5

Figure S5B

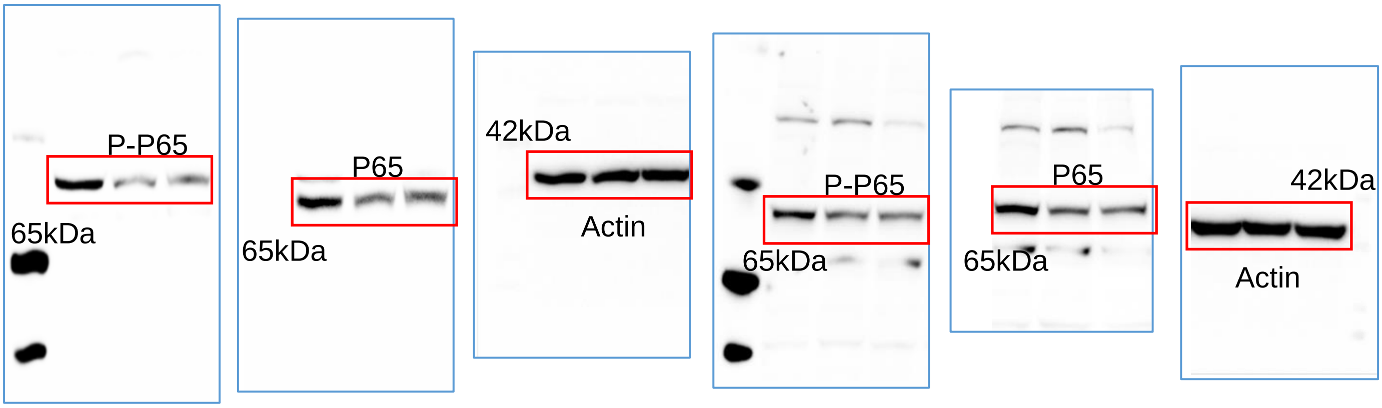

Figure S5G

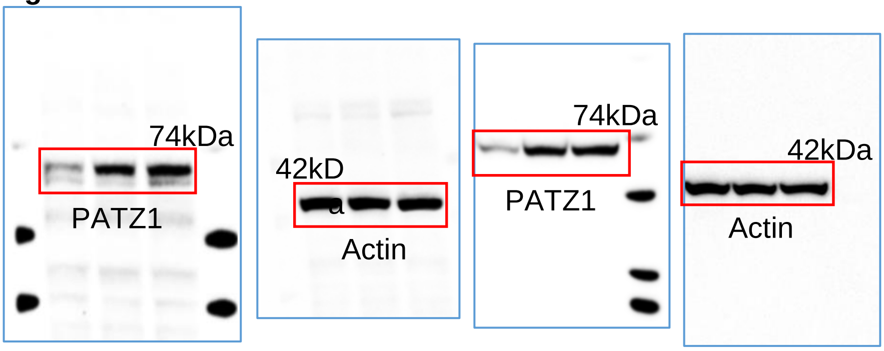

Figure S5H

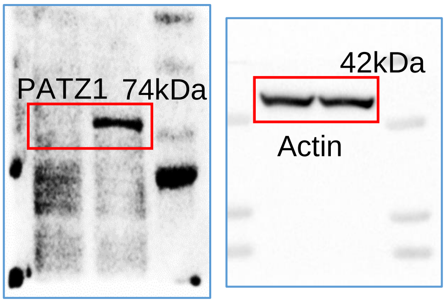

Figure S5I

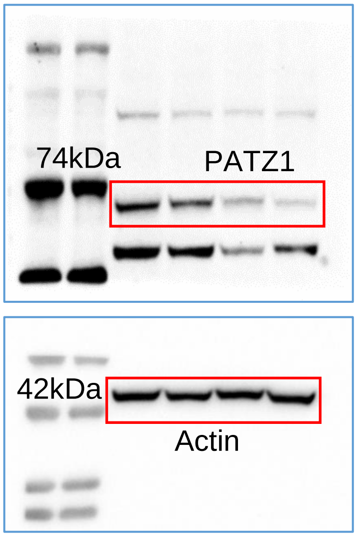

Figure S5J

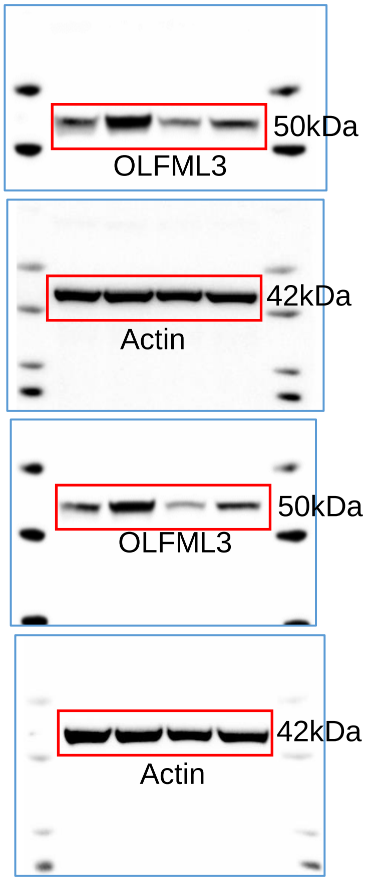

Figure S5K

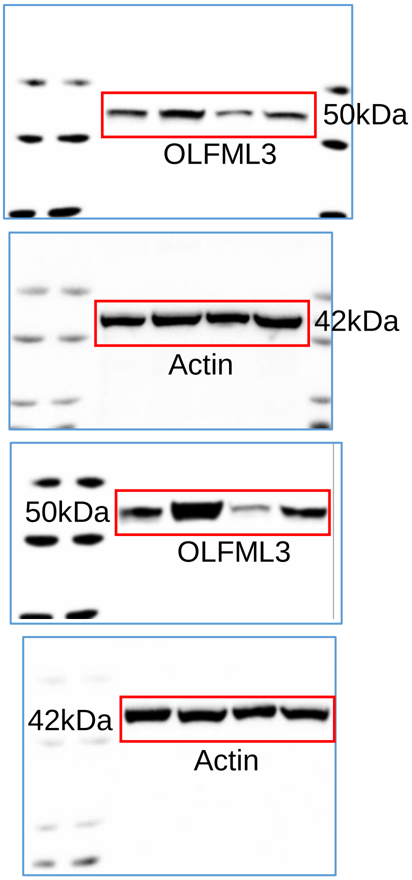

Supplement: Unedited blot and gel images [file jci-134-178628-s218.pdf]
